# A cell-autonomous role for primary cilium-mediated signaling in long-range commissural axon guidance

**DOI:** 10.1242/dev.202788

**Published:** 2024-09-05

**Authors:** Alexandre Dumoulin, Nicole H. Wilson, Kerry L. Tucker, Esther T. Stoeckli

**Affiliations:** ^1^ Department of Molecular Life Sciences, University of Zurich, Winterthurerstrasse 190, 8057 Zurich, Switzerland; ^2^ Neuroscience Center Zurich, University of Zurich, Winterthurerstrasse 190, 8057 Zurich, Switzerland; ^3^ University of New England, College of Osteopathic Medicine, Department of Biomedical Sciences, Center for Excellence in the Neurosciences, Biddeford, ME 04005, USA; ^4^ University Research Priority Program ‘Adaptive Brain Circuits in Development and Learning’ (URPP AdaBD), University of Zurich, Winterthurerstrasse 190, 8057 Zurich, Switzerland

**Keywords:** Sonic hedgehog, Axon guidance, Commissural neurons, *In ovo* RNAi, Primary cilium, Midline crossing, Ift88, Arl13b, Mouse, Chicken

## Abstract

Ciliopathies are characterized by the absence or dysfunction of primary cilia. Despite the fact that cognitive impairments are a common feature of ciliopathies, how cilia dysfunction affects neuronal development has not been characterized in detail. Here, we show that primary cilium-mediated signaling is required cell-autonomously by neurons during neural circuit formation. In particular, a functional primary cilium is crucial during axonal pathfinding for the switch in responsiveness of axons at a choice point or intermediate target. Using different animal models and *in vivo*, *ex vivo* and *in vitro* experiments, we provide evidence for a crucial role of primary cilium-mediated signaling in long-range axon guidance. The primary cilium on the cell body of commissural neurons transduces long-range guidance signals sensed by growth cones navigating an intermediate target. In extension of our finding that Shh is required for the rostral turn of post-crossing commissural axons, we suggest a model implicating the primary cilium in Shh signaling upstream of a transcriptional change of axon guidance receptors, which in turn mediate the repulsive response to floorplate-derived Shh shown by post-crossing commissural axons.

## Teaser

**Highlighted Article:** Perturbation of primary cilium function interferes with axon guidance at choice points. The resulting disturbance of neural circuit formation could explain phenotypes seen in some ciliopathy patients.

## INTRODUCTION

The primary cilium is a non-motile protrusion on the cell soma that works as a signaling hub during key developmental processes, such as survival, proliferation, differentiation, polarization and migration of cells (Goetz and Anderson, 2010). Mutations in genes that encode proteins required for primary cilia formation, maintenance or function cause a wide spectrum of human disorders, classified as ciliopathies (Reiter and Leroux, 2017). Patients have a variety of symptoms, including kidney and liver problems, limb malformations and, very often, cognitive impairments (Reiter and Leroux, 2017; Valente et al., 2014). In some types of ciliopathies, like Joubert or Bardet–Biedl syndromes, the brain is also structurally impaired, as axon tracts are aberrantly formed (Valente et al., 2014; Sattar and Gleeson, 2011). Therefore, we used animal models to better understand the etiology of these disorders by analyzing the role of the primary cilium during neural circuit formation.

Our previous results linked sonic hedgehog (Shh) signaling, a major signaling pathway associated with the primary cilium, to commissural axon guidance (Bourikas et al., 2005; Wilson and Stoeckli, 2013). Axons of the dI1 neurons extend ventrally from the dorsal spinal cord, cross the floorplate, the ventral midline, before turning rostrally along the longitudinal axis. Many guidance cues for dI1 commissural axons have been identified (Chédotal, 2011; de Ramon Francàs et al., 2017; Nawabi and Castellani, 2011; Stoeckli, 2018), including Shh, which plays multiple roles (Zuñiga and Stoeckli, 2017). Although pre-crossing axons are attracted by floorplate-derived Shh (Charron et al., 2003), axons at the midline switch their responsiveness to Shh, as post-crossing axons are repelled by Shh (Bourikas et al., 2005; Wilson and Stoeckli, 2013; Yam et al., 2012). The expression of hedgehog-interacting protein (Hhip), the receptor which is required for the repulsive response to Shh and the rostral turn of post-crossing commissural axons, is triggered by Shh itself in a glypican 1-dependent manner (Wilson and Stoeckli, 2013).

Therefore, we set out to test the requirement of the primary cilium for transcription-dependent Shh signaling during axon guidance, in analogy to what was shown previously for cell differentiation and patterning (Bangs and Anderson, 2017). There, the primary cilium was found to be essential for the transcriptional response to Shh (Nozawa et al., 2013). Binding of Shh to patched1 promotes translocation of the Shh signaling effector smoothened (Smo) to the cilium, where it activates Gli transcription factors (Corbit et al., 2005). However, in axon guidance, Shh signaling was shown to be transcription-independent (Yam et al., 2009; Toro-Tapia and Das, 2020). Similarly, transcription-independent signaling was implicated in cell migration (Bijlsma et al., 2007; Brennan et al., 2012). The subcellular localization of Smo determines the differential responses to Shh. Whereas Smo in the cilium activates Gli transcription factors to induce gene expression, Smo located outside the cilium favors the activation of chemotactic responses (Bijlsma et al., 2012).

Based on these findings and our results implicating the ciliopathy gene CPLANE1 (also termed C5ORF42 or JBTS17) in axon guidance (Asadollahi et al., 2018), we tested whether primary cilium-mediated signaling was required for Shh-dependent commissural axon guidance. To test these possibilities, we examined commissural axon guidance in the cobblestone mouse, a mouse model in which ciliary function is perturbed, and refined and extended these analyses in chicken embryos, where we could silence ciliary genes in a spatiotemporally precisely controlled manner.

Taken together, our studies indicate that a functional primary cilium is required for commissural axon guidance in a cell-autonomous manner, allowing for a transcriptional switch in responsiveness to Shh. Our data support a model of a signaling cascade triggered at the growth cone of axons crossing the floorplate that involves retrograde transport of Shh. At the soma, the signal is transduced at the primary cilium for finally inducing the transcription and expression of Hhip, the Shh receptor on post-crossing axons.

## RESULTS

### Commissural neurons carry a primary cilium before, during and after their axons cross the CNS midline

The dorsal-most subpopulation of commissural neurons (dI1 neurons) provides a very accessible neuronal population to investigate molecular mechanisms of axon guidance, as they have a very stereotypical trajectory towards and across the floorplate, the ventral midline of the spinal cord (Stoeckli, 2018).

**Fig. 1.**
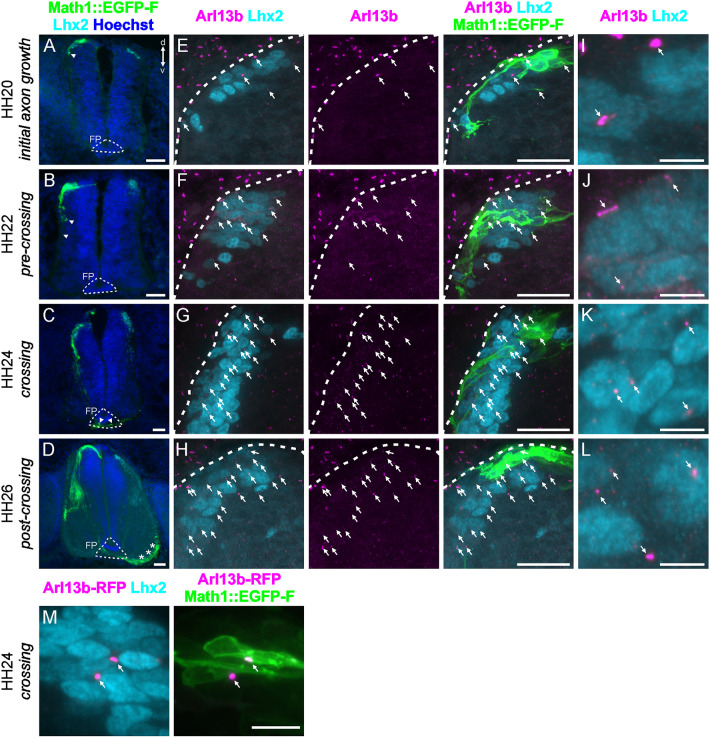
**dI1 commissural neurons carry a primary cilium during development *in vivo*.** (A-D) Math1::EGFP-F (green) electroporated at HH17-HH18 labels dI1 neurons. Axons (arrowheads) started to grow at HH20 (A), extended ventrally at HH22 (B) and completed crossing the floorplate at HH24 (C). Post-crossing axons extended in the contralateral ventral funiculus at HH26 (asterisks; D). Lhx2 is a marker for dI1 neurons (cyan). Nuclei were counterstained with Hoechst (blue). (E-H) High magnification images of A-D showing Lhx2-positive dI1 nuclei (cyan) co-stained with the primary cilium marker Arl13b (magenta) and GFP (dI1 neuron reporter, green). Neurons carried a primary cilium (arrows) on their soma throughout stages HH20 to HH26. (I-L) Magnification of areas shown in E-H. (M) Ciliation of dI1 neurons was confirmed at HH24 after co-electroporating Arl13-RFP (magenta) and Math1::EGFP-F (green) at HH17-HH18 (white arrows). FP, floorplate; d, dorsal; v, ventral. White dashed lines outline the spinal cord boundary. Scale bars: 50 µm (A-D); 25 µm (E-H); 5 µm (I-L); 10 µm (M).

**Fig. 2.**
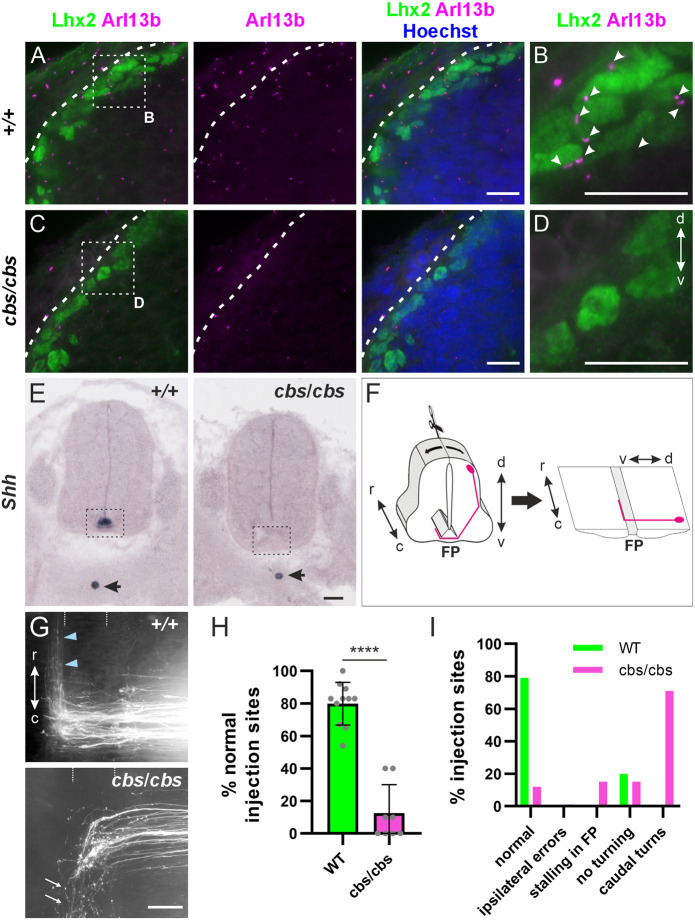
**Cobblestone mice display defects in post-crossing commissural axon guidance.** (A-D) Sections of E11.5 mouse embryos were stained for the primary cilium marker Arl13b (magenta), the dI1 neuron marker Lhx2 (green) and counterstained with Hoechst (blue). Although many primary cilia were present in the area of dI1 somas of WT embryos (+/+, arrowheads, B), almost none was detected in homozygous cobblestone (cbs/cbs) littermates (D). (E) Shh mRNA was absent from the floorplate (rectangle) in E12.5 cbs/cbs embryos, but still expressed in the notochord (arrow). (F) Schematic depicting the open-book preparation of spinal cords, allowing for the visualization of dI1 axons (magenta) at the floorplate. (G) dI1 axons in a WT E12.5 spinal cord turned rostrally (blue arrowheads), but in cbs/cbs embryos mostly aberrant caudal turns were seen (white arrows). (H) Quantification of axon guidance defects. N(embryos)=11 (WT) and 8 (cbs/cbs); *n*(injection sites)=118 (WT) and 52 (cbs/cbs). Error bars represent s.d. ****P<0.0001 (two-tailed unpaired *t*-test). d, dorsal; v, ventral; r, rostral; c, caudal; FP, floorplate. Scale bars: 20 µm (A-D); 50 µm (E,G). For data and statistics see Table S1.

We first confirmed that dI1 neurons carried a primary cilium during axon pathfinding in chicken (Fig. 1; Fig. S1) and mouse (Fig. 2) embryos. *In ovo* electroporation of a plasmid for dI1 neuron-specific expression (Math1 enhancer, Wilson and Stoeckli, 2011) together with Lhx2, as a marker for dI1 neurons, and Arl13b staining revealed the presence of primary cilia at different stages (Fig. 1A-L). Arl13b-positive cilia were found on dI1 neurons in the chicken spinal cord during the time window of axon guidance of Hamburger and Hamilton stage (HH) 20 to HH26 (Fig. 1A-L).

These findings were in line with previous reports in the chicken spinal cord with newly differentiated interneurons *in vivo* and cultured commissural neurons (Toro-Tapia and Das, 2020; Yusifov et al., 2021). At HH24, we found that 85±2% (mean±s.d.) of dI1 neurons were ciliated (*N*_embryos_=3, *n*_sections_=12, see Table S1). This suggests that most, if not all, dI1 neurons possess a primary cilium at the time when their axons cross the midline. The ciliation of dI1 neurons was confirmed by immunostaining for adenylate cyclase III (ACIII), a known marker of neuronal primary cilia (Fig. S1) (Caspary et al., 2016; Ou et al., 2009). We also overexpressed Arl13b-RFP to visualize primary cilia in the chicken spinal cord together with dI1-specific GFP expression to confirm that these neurons carried a primary cilium at the time when their axons crossed the floorplate (Fig. 1M).

### Commissural axon guidance is perturbed in the cobblestone mutant

To study the role of cilia and Shh signaling in commissural axon guidance, we examined a mouse mutant in which ciliary function is perturbed. Cobblestone (cbs) mice are hypomorphic for the intraflagellar transport protein-88 (Ift88), as they express *Ift88* mRNA and protein at only 25% of the levels of wild-type (WT) embryos (Willaredt et al., 2008). Ift88 is a component of the IFTB anterograde transport complex, which is required for formation and maintenance of cilia and transcription-dependent Shh signaling (Bangs and Anderson, 2017; Huangfu and Anderson, 2005; Liu et al., 2005). Although cbs/cbs embryos still possess cilia, albeit in reduced numbers, they phenocopy perturbations of the Shh pathway, suggesting that the existing cilia do not adequately mediate Shh signaling (Gazea et al., 2016; Willaredt et al., 2008). We not only confirmed that cbs/cbs embryos had a reduced number of cilia on dI1 neurons compared with WT littermates (Fig. 2A-D), we also found that *Shh* mRNA and protein were reduced or absent in the floorplate (Fig. 2E; Fig. S2). Hence, we used cbs/cbs mice to assess the effects of reduced Shh signaling on commissural axon guidance. In WT mice, as previously described in rat (Yam et al., 2012) and chick (Bourikas et al., 2005), *Shh* mRNA was expressed in a posterior^high^ to anterior^low^ gradient along the floorplate (Fig. S2A-D).

We assessed dI1 axon pathfinding in open-book preparations of spinal cords taken from E12.5 embryos (Fig. 2F-I). In WT or heterozygous littermates, the majority of DiI-traced axonal trajectories displayed the normal phenotype: at 80±13% of the DiI injection sites, axons crossed the floorplate and turned rostrally along the contralateral floorplate border (Fig. 2G,H). However, in cbs/cbs mice, axons at only 13±18% of the DiI injection sites showed normal behavior. Most of the axons turned caudally instead of rostrally at the floorplate exit site (Fig. 2G-I). These results suggested that aberrant Shh signaling due to cilia dysfunction caused by perturbation of Ift88 levels gave rise to aberrant commissural axon guidance.

### Cbs/cbs mice exhibit defects in ventral spinal cord patterning

Because transcription-dependent Shh signaling is required for spinal cord patterning at earlier stages, the observed axon guidance defects in the cbs/cbs mouse could also be caused indirectly through changes in floorplate induction and aberrant cell differentiation. Indeed, patterning was affected in cbs/cbs mice (Fig. S3). The characteristic distribution of Islet1-positive motoneurons was not seen in embryonic day (E)10.5 cbs/cbs mice, and Nkx2.2, a Shh target that is only induced by high levels of Shh, was strongly reduced. Shh and the floorplate marker FoxA2 (HNF3β) were absent from the cbs/cbs spinal cord (Fig. S3). In contrast, dorsal markers, like Pax3, were unaffected. Importantly, the differentiation of dI1 neurons still occurred in cbs/cbs mice, as Lhx2-positive interneurons extended their axons normally towards the ventral midline, as visualized by Axonin-1 (Cntn2) staining (Fig. S3). These results were consistent with previous reports in which no pre-crossing phenotype was seen in mice lacking other ciliary genes (Ferent et al., 2019).

As the floorplate is the intermediate target for commissural axons, its improper differentiation in cbs/cbs mice might contribute to the observed axon guidance errors. Indeed, Gli2^−/−^ mice, which lack a floorplate, display similar guidance errors to those reported here (Matise et al., 1999; Yam et al., 2012). To determine whether the axon guidance anomalies in cbs/cbs mice arose only secondarily to the lack of a defined floorplate, or whether Ift88 or ciliary function might also be directly required for correct axon guidance, we next turned to *in ovo* RNAi experiments, in which we could avoid the early morphological effects of diminished Ift88 function.

### Temporally controlled loss of the IFTB proteins Ift88 and Ift52 in the chicken spinal cord leads to axon guidance defects without affecting patterning

**Fig. 3.**
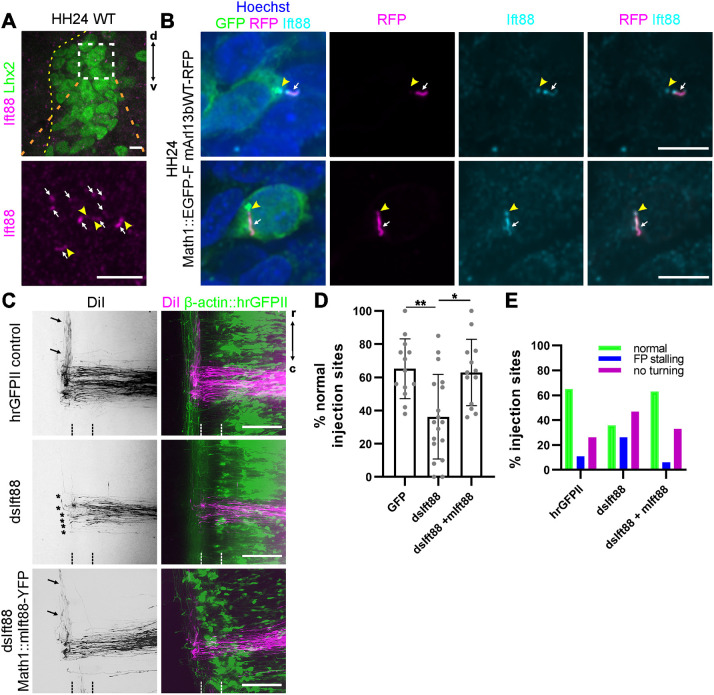
**Ift88 is cell-autonomously required for commissural axon guidance.** (A) Ift88 localized to primary cilia (white arrows) of dI1 neurons at the time when their axons cross the floorplate. Bottom panel shows magnification of boxed area in top panel (indicated by dashed orange lines). Dashed yellow line indicates spinal cord boundary. Yellow arrowheads show basal bodies. (B) Expression of the ciliary marker Arl13b-RFP in GFP-positive dI1 neurons verified accumulation of Ift88 in the cilium (white arrows) and basal body (yellow arrowhead) at the time of midline crossing. (C) Representative images of DiI-traced dI1 axons in open-book preparations of GFP-expressing control embryos, embryos lacking Ift88 (dsIft88) and embryos co-injected with dsIft88 and mouse Ift88-YFP (dsIft88; Math1::mIft88-YFP) specifically expressed in dI1 neurons. Co-electroporation of mouse Ift88 rescued normal rostral turning (black arrows). In embryos lacking Ift88, most axons failed to turn at the floorplate border (asterisks). Dashed lines mark the floorplate. (D,E) Quantification of axon guidance phenotypes. *N*(embryos)=13 (hrGFPII), 18 (dsIft88), 13 (dsIft88+mIft88-YFP); *n*(injection sites)=152 (hrGFPII), 202 (dsIft88), 171 (dsIft88+mIft88-YFP). Error bars represent s.d. *P<0.05, **P<0.01 (one-way ANOVA with Tukey's multiple comparisons test). ns, not significant (*P*>0.05). r, rostral; c, caudal; d, dorsal; v, ventral. Scale bars: 5 µm (A,B); 100 µm (C). For data and statistics see Table S1.

Using *in ovo* RNAi allowed us to precisely control Ift88 knockdown in such a way that floorplate development was not affected. *Ift88* was expressed throughout the chick neural tube during commissural axon pathfinding (HH18-HH26) (Fig. S4A,B) and Ift88 protein was found to localize to the primary cilium of dI1 neurons at the time when their axon crossed the midline (Fig. 3A,B). Unilateral electroporation of long double-stranded RNA (dsRNA) derived from *Ift88* was performed at HH17-HH18 to knockdown Ift88 in one half of the neural tube only after early spinal cord patterning was completed, but immediately before commissural axon outgrowth. The efficiency and specificity of the knockdown using dsRNA was verified using a reporter assay *in vitro* (Fig. S5). After temporally controlled silencing of *Ift88*, floorplate morphology was normal and neural tube patterning was not affected, as this was completed before Ift88 knockdown (Fig. S4C,D).

Silencing *Ift88* in this temporally controlled manner still caused axons to stall in the floorplate or at the contralateral floorplate border (Fig. 3C-E). Most of the axons failed to turn, suggesting that Ift88 was directly required for guidance of post-crossing axons. Normal turning of dI1 axons upon floorplate exit was seen only at 36±25% of the DiI injection sites. In contrast, in control-treated GFP-expressing embryos dI1 axons turned rostrally at 65±18% of the DiI injection sites. The specificity of the knockdown was demonstrated by co-electroporation of mouse *Ift88* in dI1 neurons. Mouse *Ift88* was not targeted by dsIft88 derived from chicken *Ift88*. After rescue with mouse Ift88, 63±20% of the injection sites showed normal rostral turns (Fig. 3C-E). Consistent results were found when we interfered with Ift52 function, another component of the IFTB complex that directly binds Ift88 (Lucker et al., 2010) (Fig. S6).

Taken together, the spatiotemporally controlled knock-down of the ciliary gene *Ift88* indicated that it was required cell-autonomously for correct axon guidance and that this activity was distinct from its earlier role in floorplate morphogenesis and cell differentiation (Goetz and Anderson, 2010; Tasouri and Tucker, 2011).

### Arl13b has to localize to the primary cilium for correct axon guidance

**Fig. 4.**
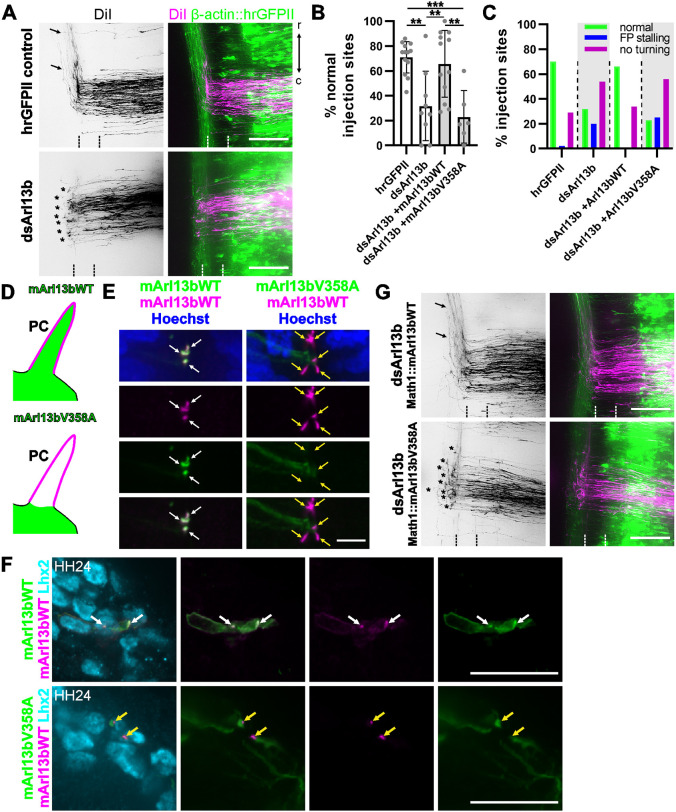
**Arl13b in the primary cilium is required for correct axon guidance.** (A) DiI-traced dI1 axons turned normally in GFP-expressing control embryos (black arrows), but not in dsArl13b-electroporated embryos (asterisks). (B,C) Quantification of axon guidance phenotypes. *N*(embryos)=8 (hrGFPII), 9 (dsArl13b), 12 (dsArl13b+mArl13bWT-GFP), 6 (dsArl13b+mArl13bV358A-GFP); *n*(injection sites)=104 (hrGFPII), 114 (dsArl13b), 163 (dsArl13b+mArl13bWT-GFP), 77 (dsArl13b+mArl13bV358A-GFP). Error bars represent s.d. **P<0.01, ***P<0.001 (one-way ANOVA with Tukey's multiple comparisons test). ns, not significant (*P*>0.05). (D) Arl13bWT-GFP can enter the primary cilium (PC), but the V358A mutant cannot. (E,F) Overexpression of either mArl13bWT-GFP or mArl13bV358A-GFP under the β-actin promoter confirmed the absence of the mutant mArl13b from mArl13b-RFP-positive cilia (yellow arrows) in the central canal at HH22 (E) and most importantly in Lhx2-positive commissural neurons at HH24 (F). (G) Rescue experiment using the dI1-specific Math1 enhancer to drive expression of mArl13bWT-GFP rescued turning of axons after silencing endogenous Arl13b, whereas mArl13bV358A-GFP did not (asterisks). Floorplate indicated by dashed lines. r, rostral; c, caudal; d, dorsal; v, ventral. Scale bars: 100 µm (A,G); 5 µm (E); 20 µm (F). For data and statistics, see Table S1.

To further investigate primary cilium signaling in dI1 axon guidance, we silenced *Arl13b*. In agreement with results seen for Ift88, the knockdown of Arl13b predominantly resulted in axons failing to turn rostrally (Fig. 4A), with normal axon trajectories at only 32±28% of DiI injection sites, compared with 70±15% in control-treated embryos (Fig. 4B,C). Importantly, *Arl13b* silencing did not induce patterning defects of the spinal cord or affect pre-crossing commissural guidance (Fig. S7).

To assess whether Arl13b is required for dI1 axon guidance in the primary cilium and in a cell-autonomous manner, we performed two types of rescue experiments. We used plasmids encoding GFP-tagged WT mouse Arl13b (Arl13bWT-GFP) or the Arl13b-V358A-GFP mutant that cannot enter the primary cilium (Fig. 4D) (Higginbotham et al., 2012). As a first step, we confirmed the *in vivo* localization of these proteins. Arl13bWT-GFP, but not the mutant protein Arl13b-V358A-GFP, effectively localized to the primary cilium of neuronal progenitors in the central canal (Fig. 4E) and to dI1 neurons (Fig. 4F). We then co-electroporated dsArl13b with these two different Arl13b constructs under the dI1-specific Math1 enhancer. Although we were able to rescue axon guidance with the expression of WT Arl13b (66±27%), the expression of Arl13bV358A did not result in rescue, with only 23±21% of injection sites displaying normal rostral turning, similar to embryos treated only with dsRNA (Fig. 4B,C,G).

Collectively, these experiments demonstrate the cell-autonomous role of Arl13b in dI1 axon guidance and the requirement for Arl13b localization to the primary cilium. Taken together with the results obtained for the interference with Ift88 function, our results demonstrate that intact primary cilium-mediated signaling is required for axonal navigation.

### Ift88 is required for the transcriptional switch of Shh receptors in commissural neurons

**Fig. 5.**
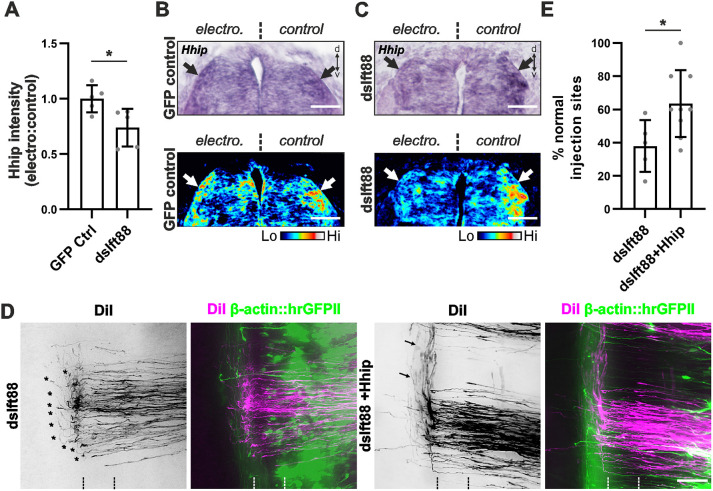
**Ift88 is required for the transcription of Hhip, the Shh receptor for post-crossing commissural axons.** (A-C) Ift88 knockdown at HH18 reduced *Hhip* mRNA expression (arrows in B,C) in dI1 neurons at HH24 on the electroporated compared with the control side by ∼25%. Electroporation of a GFP-expressing plasmid had no effect (A,B). *N*(embryos)=5 for each condition. Hhip mRNA expression was quantified by comparing the electroporated versus the control side because levels differ strongly along the spinal cord. Therefore, ratios were compared between experimental and control embryos rather than absolute values. Lower row (B,C) shows color-coded mRNA levels of the images shown in the upper row. (D,E) Axon guidance errors seen after Ift88 downregulation were rescued by Hhip expression. (D) dI1 axons failed to turn rostral when Ift88 was silenced (asterisks), but concomitant expression of mouse Hhip rescued the turn (arrows). (E) The number of DiI injection sites with normal axonal trajectories were significantly increased compared with dsIft88 and similar to GFP controls (see Fig. 3D, two-tailed unpaired *t*-test). *N*(embryos)=5(dsIft88) and 9(dsIft88+Hhip); *n*(injection sites)=62 (dsIft88) and 79 (dsIft88+Hhip). Error bars represent s.d. *P<0.05 (two-tailed unpaired *t*-test). electro, electroporated; Hi, high; Lo, low. For data and statistics, see Table S1.

We previously identified an Shh-mediated and glypican 1-dependent trigger of *Hhip* transcription as responsible for the change from attraction to repulsion between pre- and post-crossing commissural axons (Wilson and Stoeckli, 2013). Because the phenotypes observed after silencing *Ift88* were similar to those seen after the perturbation of Shh, glypican 1 or Hhip function (Bourikas et al., 2005; Wilson and Stoeckli, 2013), we hypothesized that the axon misprojections observed after *Ift88* silencing could be caused by the absence of Hhip expression. Indeed, when Ift88 was unilaterally knocked down, *Hhip* mRNA signal intensity in the dI1 neurons (Fig. 5) was lower on the electroporated side (Fig. 5C). As expected, electroporation of a plasmid encoding GFP (control) did not reduce *Hhip* expression (Fig. 5B). Silencing *Ift88* significantly reduced *Hhip* mRNA by about 26% compared with the non-electroporated side with a ratio^electro:control^ of 0.74±0.17 compared with 1±0.12 for the control sample (Fig. 5A). The reduction in Hhip expression explains the failure of post-crossing commissural axons to turn rostrally (Bourikas et al., 2005; Wilson and Stoeckli, 2013).

To provide additional evidence supporting our model that Ift88 function is required for Shh-mediated induction of *Hhip* expression, and thus, repulsion of post-crossing axons, we carried out rescue experiments. Consistent with our hypothesis, restoring Hhip expression in dI1 neurons lacking Ift88 resulted in normal axon guidance. We found normal axonal navigation at 63.5±20% of the DiI injection sites (Fig. 5D,E). This was not significantly different from control-treated embryos (see Fig. 3D). In comparison, after knocking down Ift88, we found normal axon guidance at only 38.0±16% of the injection sites.

Taken together, the reduction of *Hhip* expression after Ift88 knockdown and the rescue of the Ift88-dependent axon guidance errors by restoring Hhip expression support the idea that Ift88 is part of the Shh-glypican 1-Hhip signaling cascade by acting upstream of Hhip.

### Live imaging of dI1 commissural axons *ex vivo* suggests a link between Smo localization in primary cilia and rostral turning of commissural axons

**Fig. 6.**
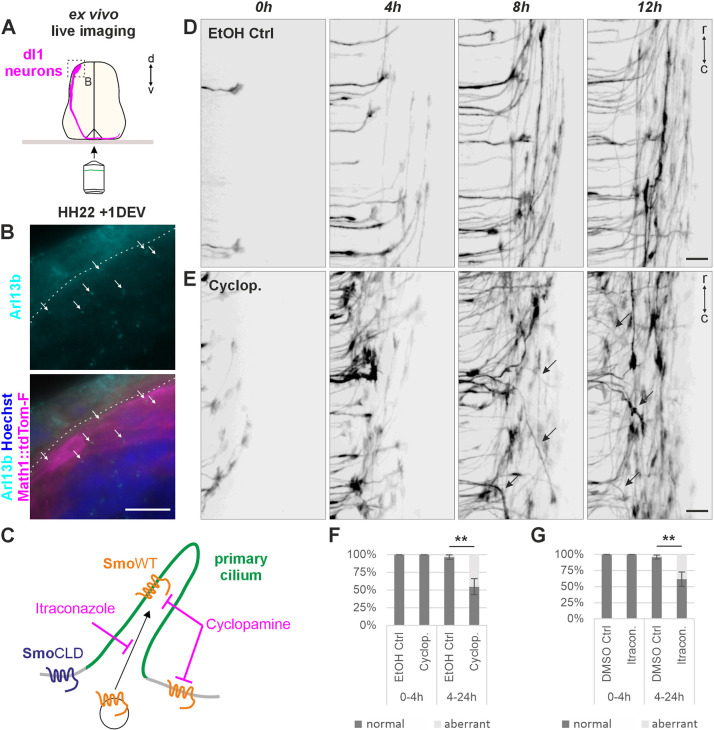
***Ex vivo* live imaging suggests a link between Smo localization in primary cilia and rostral turning of commissural axons.** (A) Schematic depicting the *ex vivo* set-up for visualization of dI1 axon navigation in the intact spinal cord. (B) After 24 h of *ex vivo* culture, primary cilia were stained with Arl13b (white arrows, cyan) on dI1 neuron cell bodies (magenta). Nuclei counterstained with Hoechst (blue). (C) Schematic of *ex vivo* smoothened (Smo) inhibition with Cyclopamine (general inhibition of Smo) and Itraconazole (inhibition of Smo entry into the primary cilium). The mutant SmoCLD cannot enter the primary cilium. (D,E) In the presence of 15 µM Cyclopamine (Cyclop) axons failed to turn rostrally at the floorplate exit site (black arrows, E). Ethanol (EtOH) was used as vehicle control (D). (F,G) Quantification of the axon guidance phenotype in the presence or absence of Cyclopamine or Itraconazole (Itracon). No (0%) aberrant phenotype was seen in the first 4 h of imaging in all conditions. However, between 4 and 24 h of imaging, significant and similar increases of aberrant phenotypes for both inhibitors were found compared with controls. *N*(embryos)=3 for each condition; *n*(axons)=19 (ethanol Ctrl^0-4h^), 36 (Cyclopamine^0-4h^), 198 (ethanol Ctrl^4-24h^), 184 (Cyclopamine^4-24h^), 39 (DSMO Ctrl^0-4h^), 40 (Itraconazole^0-4h^), 142 (DSMO Ctrl^4-24h^) and 234 (Itraconazole^4-24h^). Error bars represent s.d. **P<0.01 (two-tailed unpaired *t*-test), DEV, day *ex vivo*; r, rostral; c, caudal. Scale bars: 10 µm (B); 20 µm (D,E). For data and statistics, see Table S1.

To further support the implication of primary cilia in this pathway, we used our previously described *ex vivo* culture system to visualize dI1 axon behavior in real time (Fig. 6A) (Dumoulin et al., 2021). We first confirmed that dI1 neurons still carried a primary cilium after 1 day *ex vivo* (Fig. 6B).

Transcriptional output of primary cilium-dependent Shh signaling involves Smo translocation into the cilium (Briscoe and Thérond, 2013). Thus, we took advantage of the *ex vivo* culture system and applied pharmacological blockers to inhibit Smo function during midline crossing (Fig. 6C). The presence of Cyclopamine in the culture medium blocked Smo function inside and outside the primary cilium (Fig. 6C). Under these conditions, post-crossing dI1 axons either failed to turn or randomly turned into the longitudinal axis, with a considerable number of axons turning caudally instead of rostrally (Fig. 6E), thus forming a disorganized post-crossing segment in comparison with the ethanol-treated control samples (Fig. 6D; Movie 1). Remarkably, the Cyclopamine-mediated inhibition of Smo did not induce instantaneous aberrant axon guidance. There was no obvious effect on commissural axons exiting the floorplate during the first 4 h of inhibition (Fig. 6E,F; Movie 1). In fact, dI1 growth cones that were traversing the floorplate, or were about to exit it, when the inhibitor was added, all turned normally at the contralateral border. Most of the axons that were not in the floorplate at the beginning of live imaging showed aberrant phenotypes (Movie 2). Between 4 and 24 h of recording, the number of axons that turned caudally or stalled at the floorplate exit site was significantly higher (45±11%) than in control (3±3%; Fig. 6F). The fact that the Cyclopamine-mediated Smo inhibition did not have an immediate effect on axon guidance was in agreement with our model that the aberrant phenotypes were caused by a transcriptional function of Smo. This also suggested that these phenotypes were not due to a growth cone-localized function of Smo as described for pre-crossing commissural axons (Yam et al., 2009; Toro-Tapia and Das, 2020). In line with our hypothesis, inhibition of Smo entry into the cilium with Itraconazole (Fig. 6C) (Kim et al., 2010) resulted in a very similar outcome. Axons behaved normally for the first 4 h of culture, with 100% of axons turning rostrally (Fig. 6G), but then, a significant number of axons (38±8%) turned caudally or stalled at the contralateral floorplate border compared with the vehicle-treated control (4±2%, Fig. 6G; Movie 3).

Taken together, the real-time monitoring of axon guidance combined with pharmacological blockers suggest a function of Smo outside the growth cone but inside the primary cilium.

### Smo localization in the primary cilium is required for the induction of *Hhip* transcription and correct dI1 axon guidance *in vivo*

**Fig. 7.**
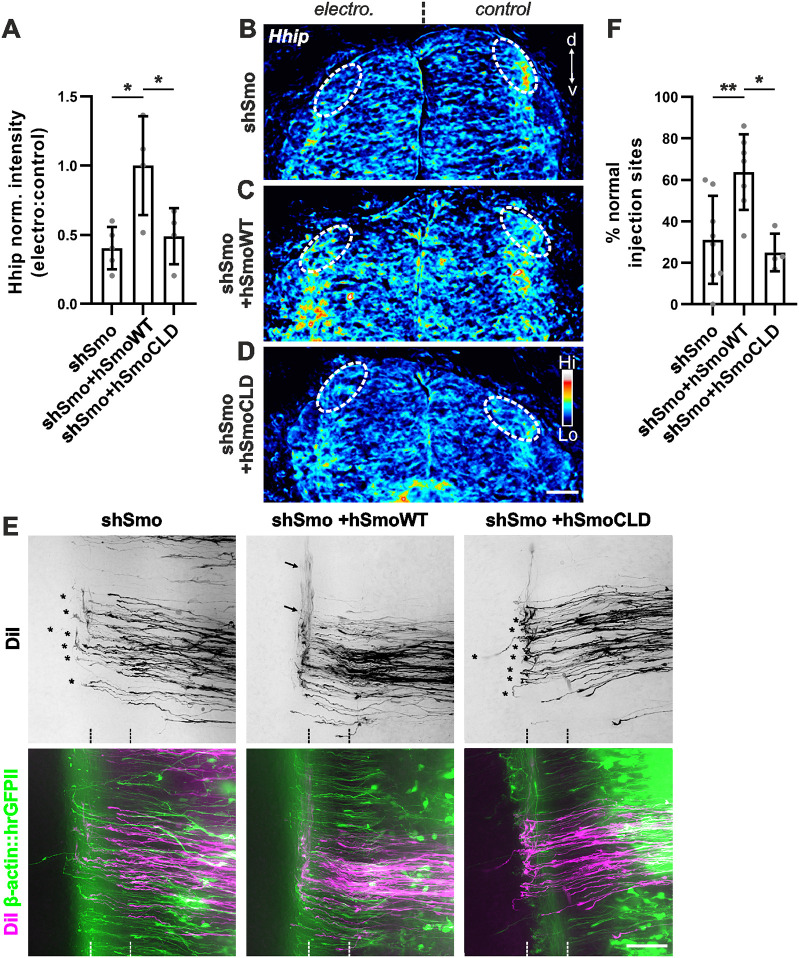
**Smo in the primary cilium is required for *Hhip* transcription in dI1 neurons and correct axon guidance *in vivo*.** (A) Quantification of *Hhip* mRNA in dI1 neurons represented as the ratio between electroporated and control side and normalized to the average ratio of the shSmo+hSmoWT condition. *N*(embryos)=5 (shSmo), 4 (shSmo+hSmoWT) and 4 (shSmo+hSmoCLD). Each dot represents the average normalized ratio per embryo. (B-D) Heat-map of HH24 spinal cords showing *Hhip* mRNA. Co-electroporation of WT human Smo (hSmoWT; C), but not SmoCLD (D), rescued *Hhip* expression (dashed ovals) that was lost after Smo knockdown (B) compared with the control side (C). (E,F) Rescue of *Hhip* expression after downregulation of Smo by co-electroporation of hSmoWT rescued shSmo-induced axon guidance errors. Co-electroporation of hSmoCLD was not able to rescue axon guidance (E). Asterisks and arrows show dI1 axonal growth cones stuck at the floorplate exit site and dI1 axons that correctly turn rostral, respectively. (F) Quantification of the percentage of aberrant phenotypes (mean±s.d.). *N*(embryos)=8 (shSmo), 7 (shSmo+hSmoWT) and 4 (shSmo+hSmoCLD). *P<0.05, **P<0.01 (one-way ANOVA with Tukey's multiple comparisons test). ns, not significant (*P*>0.05). Hi, high; Lo, low; electro, electroporated. Scale bars: 50 µm. For data and statistics, see Table S1.

We previously showed that the expression of a constitutively active Smo in the spinal cord upregulated *Hhip* mRNA expression in dI1 neurons, suggesting a role for Smo in the Shh-glypican 1-Hhip signaling pathway upstream of Hhip (Wilson and Stoeckli, 2013). For further support of the role of Smo in this pathway, we investigated *Hhip* expression in embryos in which Smo localization to the cilium was perturbed. To achieve this, we knocked down endogenous chicken Smo, using shRNA (Fig. S8), and then attempted to rescue Hhip expression by co-electroporating constructs encoding WT human Smo (hSmoWT) or a Smo mutant with two amino acid substitutions that prevent ciliary localization (hSmoCLD). In contrast with hSmoWT, hSmoCLD cannot activate the transcription-dependent Shh response (Bijlsma et al., 2012; Corbit et al., 2005). Supporting the idea that *Hhip* induction requires the cilium, we found that hSmoWT, but not hSmoCLD, could rescue *Hhip* expression in dI1 neurons following knockdown of endogenous Smo (Fig. 7). The average normalized ratio of *Hhip* expression in dI1 neurons after rescue with hSmoWT was significantly higher than that found after either knockdown or rescue attempt with hSmoCLD (Fig. 7A-D). Furthermore, expression of hSmoWT, but not hSmoCLD, rescued the axon guidance phenotypes induced by silencing endogenous *Smo* (Fig. 7E). After silencing *Smo* and rescue with hSmoWT, normal axon guidance was seen at 64±17% of the DiI sites, compared with only 31±20% (shSmo) and 25±8% (shSmo+hSmoCLD) (Fig. 7F).

Importantly, Smo silencing with shRNA did not affect patterning or pre-crossing commissural axon growth (Fig. S9). Because *Hhip* induction in the dorsal spinal cord and post-crossing axon navigation rely on the ability of Smo to localize to the cilium, these results implicate primary cilium-mediated Shh signaling in axonal navigation.

### Shh is transported from the axons to the soma of commissural neurons

When commissural axons cross the midline, Shh is exclusively expressed in the floorplate (Bourikas et al., 2005). The requirement for the ciliary genes *Ift88 and Ift52*, as well as Smo localization to the primary cilium to trigger the Shh-glypican 1-Hhip signaling cascade, in dI1 neurons implies retrograde Shh transport from the growth cone to the soma, where the primary cilium is localized.

**Fig. 8.**
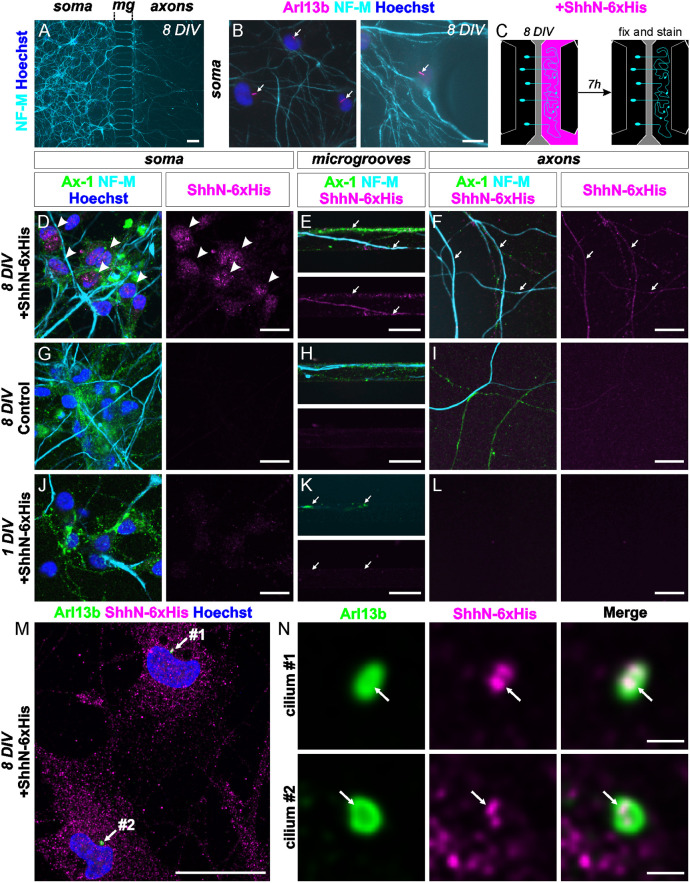
**Shh is transported from the growth cone to the soma of commissural neurons where it can reach their primary cilium in microfluidic cell cultures.** (A) Commissural axons stained with neurofilament-M (NF-M; cyan) cultured for 8 days in compartmentalized microfluidic chambers reached the ‘axon compartment’. (B) Neurons still carried a primary cilium (white arrows), visualized by Arl13b staining (magenta). (C) Schematic of the experimental setup used to assess the localization of ShhN tagged with 6xHis (ShhN-6xHis, magenta). (D-L) After 8 days, ShhN-6xHis was added for 7 h to the ‘axon compartment’. Neurons were stained for neurofilament-M (cyan), commissural neuron (CN) marker Axonin-1 (Ax-1; green) and His tag (magenta). (D-F) Shh (magenta) could be seen in the ‘axon compartment’, in microgrooves (mg; white arrows) and at the soma (white arrowheads). (G-I) No His staining was seen without adding recombinant Shh. (J-L) Most importantly, after only 1 day, when axons had not yet reached the ‘axon compartment’ (white arrows point to growth cones traversing the microgrooves at the time of fixation in K), there was no Shh in the ‘soma compartment’. This confirmed the proper fluidic compartmentalization between chambers. (M,N) Arl13b co-staining with ShhN-6xHis in commissural neurons cultured for 8 DIV as shown in D revealed the localization of ShhN at the primary cilium (arrows). Nuclei were counterstained with Hoechst. DIV, days *in vitro*. Scale bars: 100 µm (A); 10 µm (B); 20 µm (D-M); 1 µm (N).

To investigate this aspect, we used microfluidic chambers with two chambers separated by microgrooves to culture dissociated commissural neurons. Neurons were seeded in the ‘soma chamber’ and cultured for 8 days to allow axons to reach the ‘axon chamber’ (Fig. 8A). With this experimental setup, we could successfully ‘separate’ commissural axons from their soma. Importantly, after such a long time in culture, commissural neurons still carried a primary cilium (Fig. 8B). To assess whether Shh could be transported from the axons to the soma of commissural neurons, we added ShhN-6xHis to the ‘axon chamber’. Indeed, we detected Shh at the axonal level in the ‘axon compartment’ (Fig. 8C), in the microgrooves between chambers and also at the soma level (Fig. 8D-F). We could not see any signal in control samples where no Shh was added (Fig. 8G-I). Moreover, in microfluidic chambers containing commissural neurons that were cultured for only 1 day (Fig. 8J-L), and which therefore did not yet have axons reaching the ‘axon chamber’ (Fig. 8K), we did not see Shh at the soma level (Fig. 8J). This means that both ‘soma’ and ‘axon chambers’ were fluidically isolated from each other during the 7 h incubation time. Therefore, we can be confident that the ShhN-6xHis that we found localized to the primary cilium on the cell bodies of commissural neurons in these cultures was transported there via the axons (Fig. 8M,N). Additional confirmation of Shh transport along the axons was provided by co-cultures of commissural neurons with HEK cells producing Shh-mRuby3 in the ‘axonal compartment’ (Fig. S10A). Importantly, immunostaining allowed us to visualize ShhN-6xHis localization to the primary cilium of commissural neurons in those cultures (arrows, Fig. 8M,N) demonstrating that Shh transported along commissural axons can reach the signaling hub, the cilium.

**Fig. 9.**
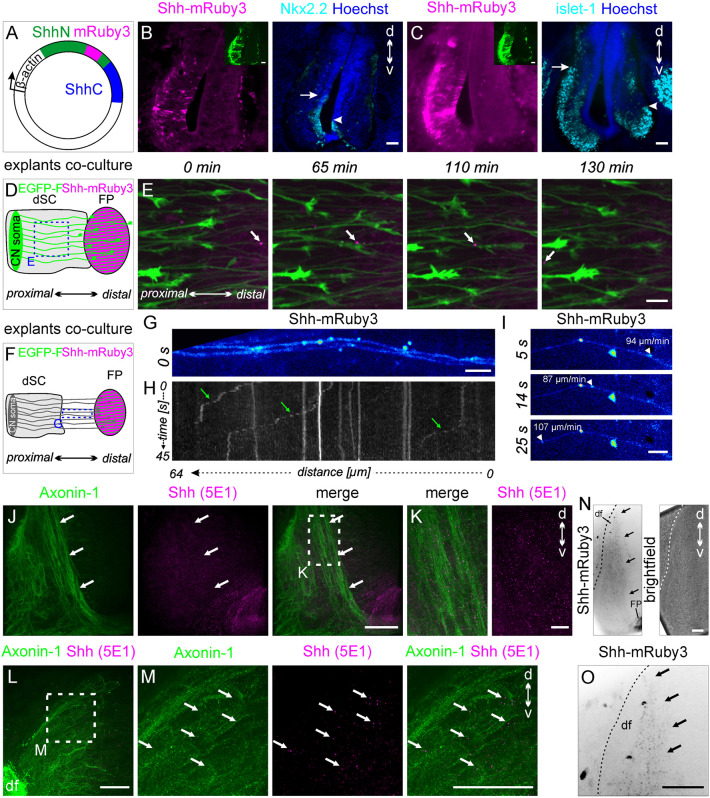
***In vitro* live imaging and *in vivo* visualization of Shh further indicate a retrograde transport of Shh to the soma of commissural neurons.** (A) Schematic of the plasmid containing the mRuby3 sequence, inserted before the end of the ShhN sequence. (B,C) Overexpression of Shh-mRuby at an early stage induced ‘ventralization’ of the spinal cord, with dorsal expansion of Nkx2.2 and Islet-1 expression (arrows) on the electroporated compared with the control side (arrowheads), demonstrating that Shh-mRuby was active. (D) Schematic of co-cultures of dorsal spinal cord (dSC; green due to Math1::EGFP-F expression) and floorplate explants expressing Shh (β-actin::Shh-mRuby3). (E) Shh-mRuby3-positive particle (white arrow) moving retrogradely along dI1 axons. (F) Schematic of co-cultures placed at a distance to better visualize axons growing directly on the dish (blue rectangle). (G) Snapshot of a time-lapse recording of Shh-mRuby3 (Movie 5). (H) Kymographic analysis revealed Shh-mRuby3-positive particles moving retrogradely along axons (green arrows). (I) Three frames extracted from the time-lapse recording shown in Movie 6. The average particle speed is shown at each time point (arrowheads). (J-M) Endogenous Shh staining *in vivo* revealed its localization along Axonin-1-positive pre-crossing axons (arrows, J,K) and in the soma of commissural neurons at HH23.5 (arrows M). (N,O) This was confirmed by visualizing endogenous fluorescence of Shh-mRuby3 particles secreted by the floorplate *in vivo* that reached the most dorsal part of the spinal cord (arrows). The boundary of the spinal cord is shown with dashed lines in N and O. d, dorsal; df, dorsal funiculus; FP, floorplate; v, ventral. Scale bars: 50 µm (B,C,J,N,O); 20 µm (E,L,M); 10 µm (K); 5 µm (G,I).

Although the experiments with microfluidic chambers were very artificial, they allowed us to confirm the long-range transport of Shh. To confirm this in a more *in vivo*-like situation, we co-electroporated a plasmid encoding chicken Shh fused to mRuby3 under the β-actin promoter (Fig. 9A). The activity of Shh-mRuby3 was confirmed by unilateral electroporation at an early stage (E2) (Fig. 9B,C). In co-cultures of floorplate and dorsal spinal cord explants (Fig. 9D), we observed Shh-mRuby3-positive particles retrogradely moving along dI1 axons (Fig. 9E). Although it was challenging to follow these particles for a long period due to photobleaching, we could clearly visualize some of these particles reaching the soma of dI1 neurons (Movie 4).

For better visualization of the retrograde Shh transport, we repeated the experiment with explants that were placed further apart, so that axons grew on the surface of the dish before contacting the floorplate cells (Fig. 9F). This allowed for better tracking of Shh-mRuby3 with live imaging (Fig. 9G-I; Movies 5,6). Using this setup, we could visualize the transport of Shh-mRuby3 to the cell body (Fig. 9G) in a stop-and-go manner (Fig. 9H). The speed of Shh-mRuby3 along axons (20-160 µm/min) was compatible with fast retrograde axonal transport (Fig. 9I). Retrograde transport to the dI1 cell body was confirmed *in vivo* by staining endogenous Shh with the 5E1 antibody (Fig. 9J-M). Shh was seen along the pre-crossing axon bundle (Fig. 9J-K) and in the area of the cell bodies (Fig. 9L,M; Fig. S10B-E). Finally, the expression of Shh-mRuby in the floorplate indicated that Shh can reach the neuronal cell body *in vivo* (Fig. 9N,O).

Taken together, our experiments support a model in which Shh signals at the primary cilium to induce transcription of *Hhip*, its own receptor for the post-crossing phase of axon guidance. Shh is taken up from the floorplate upon contact of the growth cone with the floorplate and transported to the primary cilium on the cell soma.

## DISCUSSION

Genes related to primary cilia formation and function play crucial roles during development of the nervous system at many levels (Asadollahi et al., 2018; Green et al., 2018; Guo et al., 2019; Hasenpusch-Theil and Theil, 2021; Park et al., 2019; Suciu and Caspary, 2021; Tadenev et al., 2011). Here, we used mouse and chicken embryos to investigate the role of the primary cilium in axon guidance. The use of the cobblestone (cbs/cbs) mouse, a hypomorphic mouse model of the IFTB gene *Ift88*, with reduced ciliation of dI1 neurons, revealed axon guidance defects at the floorplate (Fig. 2). Although these observations were compatible with a role for primary cilia in regulating the guidance of dI1 axons at the intermediate target, the ventral patterning defects and loss of Shh in the floorplate in this mouse mutant precluded any clear conclusion. However, thanks to the spatiotemporal precision of *in ovo* RNAi-mediated knockdown of Ift88 in the chicken spinal cord, we could demonstrate a cell-autonomous role for Ift88 in axon guidance (Fig. 3). The observed phenotype was compatible with a loss of axonal sensitivity to the Shh gradient that is driving post-crossing axons rostrally towards lower Shh levels (Bourikas et al., 2005). In contrast to the mouse, where Ift88 reduction interfered with cell differentiation (Fig. S3), the temporally controlled downregulation of Ift88 in chicken embryos did not affect cell differentiation, nor growth rate or guidance of pre-crossing axons (Fig. S4). Instead, we specifically found an effect on post-crossing axons (Fig. 3; Fig. S6). Ift88 was found to function upstream of Hhip, regulating its transient expression and engagement in Shh-mediated axon guidance (Fig. 5). Similar results were obtained with temporally controlled downregulation of Arl13b, which had to localize to the primary cilium to ensure correct axonal navigation (Fig. 4).

**Fig. 10.**
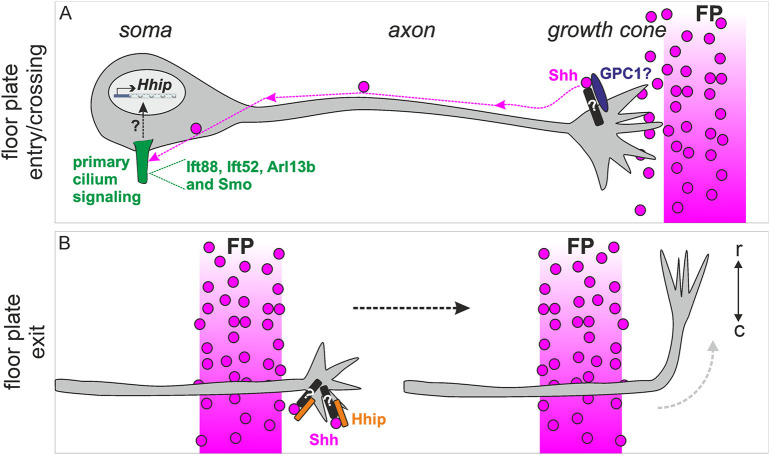
**The primary cilium is required for the regulation of *Hhip* transcription by Shh and, therefore, the switch in axon responsiveness at the floorplate.** (A) Shh produced by the floorplate is retrogradely transported to the soma of commissural neurons, when their growth cones contact the intermediate target. Glypican 1 interacts with Shh at the growth cone and, by a yet unknown mechanism, Shh is transported retrogradely to the soma. A functional primary cilium and Smo localization to the cilium are required for commissural axon guidance. (B) Once commissural axons exit the floorplate, Hhip mediates the repulsive response to the caudal^high^-rostral^low^ Shh gradient and the rostral turn. Black rods represent unknown co-receptors. FP, floorplate; GPC1, glypican 1; r, rostral; c, caudal.

Because the ciliary protein Arl13b has been reported to play a role outside the cilium in the guidance of pre-crossing dI1 axons in a transcription-independent manner in the mouse (Ferent et al., 2019), we could not *a priori* rule out a cilium-independent role for Ift88. However, we would argue that this is different for post-crossing axons. Our studies demonstrate that the primary cilium is required for *Hhip* transcription and that Smo needs to localize to the cilium for correct axon guidance (Figs 5–7). Thus, our data suggest a role for the primary cilium in switching the responsiveness of commissural dI1 axons to Shh from attraction to repulsion at the intermediate target (Fig. 10). This implies a switch of Shh signaling in these neurons from a transcription-independent to a transcription-dependent manner (Fig. 10) (Yam et al., 2012; Toro-Tapia and Das, 2020). The average time dI1 axons take from their first contact with the floorplate (Shh-producing cells) to their turning at the contralateral border (∼7 h) is compatible with a transcriptional switch of Shh receptors from Boc/Patched to Hhip (Dumoulin et al., 2021).

Our results indicate that Shh is indeed transported retrogradely after dI1 axons reach the floorplate, the only Shh source in the spinal cord at that time. The cilium on the cell body is located 400-500 µm away from the growth cone and the floorplate. Our microfluidic assays and co-culture experiments revealed retrograde Shh transport and thus supported this hypothesis (Figs 8,9).

Shh was shown to be anterogradely transported along retinal ganglion cell axons and to be secreted in the optic chiasm, where it could act as a repellent for ipsilateral populations of axons (Peng et al., 2018). Here, our results show that dI1 axons retrieve and transport Shh in a retrograde manner.

Future experiments will be required to assess whether Shh is transported on the surface of dI1 axons or whether it is internalized. By analogy to other systems, we could imagine two plausible scenarios: (1) Shh could be internalized at the growth cone, transported internally and secreted at the soma, as is the case in Shh transcytosis in epithelial cells (Ho and Stearns, 2021), or (2) as an interaction between glypican 1 and Shh is required in our system, we can imagine that Shh is transported retrogradely on the surface of dI1 axons, as is the case in *Drosophila* with the glypican 1 orthologue Dally-like protein, which is present in cytonemes of receiving cells (González-Méndez et al., 2017). In our case, dI1 axons might play the role of a receiving cytoneme for long-range transport of Shh to the neuronal soma (Fig. 10A).

In summary, our model proposes that floorplate-derived Shh is retrogradely transported by dI1 axons after contact with their intermediate target. Shh reaches their soma and transmits a signal at the primary cilium, which induces *Hhip* transcription (Fig. 10A). When axons exit the floorplate, Hhip is expressed on the growth cone surface and acts as an Shh receptor to respond to the repulsive Shh gradient and make the axon turn rostrally (Fig. 10B).

A role of the primary cilium in axon guidance might contribute to our understanding of the etiology of human ciliopathies. The results presented here are in agreement with our findings on the role of CPLANE1 in neural circuit formation (Asadollahi et al., 2018). Most importantly, the axon guidance phenotypes observed in chicken embryos after silencing the ciliopathy gene CPLANE1 were very similar to those described here. Interestingly, some *Arl13b* mutations identified in individuals with Joubert syndrome have been shown to hinder Arl13b localization to the primary cilium (Cantagrel et al., 2008; Mariani et al., 2016). Taken together, these studies suggest that signaling at the primary cilium is required to switch axonal responsiveness to a choice point during axon guidance.

## MATERIALS AND METHODS

### Animals

All experiments were approved and carried out according to the guidelines of the Cantonal Veterinary Office Zurich (ZH089/16). Cobblestone (cbs/cbs) mice were generated as previously described (Willaredt et al., 2008) and genotyped by PCR, using genomic DNA with the following primer sets: forward1, 5′-TTGACATCTGGATATGACAATGC; reverse1, 5′-TGTGCATGTTTGTGTACATATGTG; forward2, 5′-TGGTGTCTCCTTCGGAATTT; reverse2: 5′-TAAATGTAAAAGGTAAAGGCAATGG. Noon of the day of discovery of the vaginal plug was designated E0.5. Fertilized chicken eggs were obtained from a local farm (Brüterei Stöckli, Ohmstal, Switzerland) and staged according to Hamburger and Hamilton (Hamburger and Hamilton, 1951).

### Assessment of the longitudinal Shh gradient

WT E12.5 NMRI mice were killed, pinned flat and fixed in 4% paraformaldehyde (PFA) in phosphate buffered saline (PBS) for 2 h at room temperature, before rinsing in PBS, and soaking in 25% sucrose in PBS overnight. Embryos were frozen in TissueTek O.C.T. compound. On each slide, ten 25 µm-thick transverse sections were collected at 400 µm intervals. The collected area spanned from the hindlimbs to the forelimbs. After *in situ* hybridization (ISH), *Shh* intensity in the floorplate was calculated using ImageJ software (National Institutes of Health). *In situ* images were inverted, the floorplate area was selected, and the mean gray value was measured. The selected border was moved to an area in the dorsal spinal cord (where no *Shh* is present) and the mean gray value was resampled as a background measurement. *Shh* measurements along the longitudinal axis were normalized within each embryo by dividing the measured intensity by the value obtained in the hindlimb region. Thus, normalized *Shh* intensity values of <1 in the mid-trunk and forelimb levels indicated weaker expression than in the hindlimb region. Twelve embryos were used in the analysis. Data are presented as mean±s.e.m. and were subjected to a single-sample *t*-test against an intensity of 1 (arbitrary units).

### Long dsRNA and siRNA

Chicken expressed sequence tags (ESTs) of *Ift88* (ChEST 972d21), *Ift52* (ChEST 490p6), and *Arl13b* (ChEST 159n3) were obtained from Source BioScience. The *Ift88* plasmid was linearized with EcoRI or NotI, the *Ift52* plasmid with KpnI or SacI, and the *Arl13b* plasmid with EcoRV or NotI (New England Biolabs) for the synthesis of sense and antisense single-stranded RNAs (ssRNA) with T7 and T3 RNA polymerase (Promega), respectively. Equal amounts of purified ssRNAs were annealed by allowing the solution to cool slowly to room temperature after heating at 95°C for 10 min. Successful double-strand formation was verified by gel electrophoresis. Long dsRNAs were electroporated at a concentration of 400 ng/µl in PBS, together with 40 ng/µl of a reporter plasmid encoding humanized *Renilla* GFP (hrGFPII, Stratagene) and 0.01% (w/v) Fast Green (AppliChem).

Effectiveness of target gene silencing was calculated in comparison with reporter plasmid expression (Figs S5,S8). The *Ift88* or *Ift52* EST sequences were cloned into a CMV-driven vector, between the stop codon of *EGFP* and a poly-A tail. Long dsRNAs were digested to siRNAs and purified according to the instructions of ShortCut^®^ RNase III (New England Biolabs). COS7 cells were co-transfected with the Ift reporter constructs, together with EBFP2 (as a transfection control) and siRNA against *RFP*, *Ift52* or *Ift88*, using Lipofectamine 2000 (Invitrogen) in LabTekII chamber slides (Nunc). Expression levels of EBFP2 and EGFP were assessed 24 h later. Ten images in each condition were quantified in ImageJ (mean gray values), normalized to transfection levels (EGFP:EBFP2) and subjected to statistical analyses (one-way ANOVA with Tukey's multiple comparisons test).

### Cloning of shRNAs for the knockdown of smoothened

Plasmids encoding shRNAs against chicken *Smo* and Luciferase were synthesized as previously described (Wilson and Stoeckli, 2011). The target sequences were: (shSmo) 5′-AAGTGCAGAACATCAAGTTCA; (shLuc, against firefly Luciferase; Wilson and Stoeckli, 2011) 5′-CGTGGATTACGTCGCCAGTCAA. For *in ovo* electroporations, we used plasmids (250 ng/µl) containing a β-actin promoter to drive the expression of hrGFPII and an shRNA, followed by a poly-A tail in PBS.

The effectiveness of the shRNAs was tested indirectly using a similar method to that described above for dsRNAs (Fig. S8; Wilson and Stoeckli, 2011). shRNAs were cloned into pRFPRNAiC vectors and co-transfected into COS7 cells along with *Smo* reporter constructs, in which a 2.2 kb fragment of chick *Smo* was cloned downstream of EGFP in either the 5′-3′ direction or, as a negative control, in the opposite orientation. Expression levels of RFP and EGFP were assessed 24 h later. Fifteen images in each condition were quantified in ImageJ (mean gray values), normalized to transfection levels (EGFP:RFP) and subjected to statistical analyses (unpaired *t*-test).

### SmoWT and SmoCLD constructs

A cDNA clone containing human *SMO-M2*, a constitutively active form of human *SMO* with a single point mutation (W535 L) was kindly provided by J. Briscoe (The Francis Crick Institute, London, UK). This construct was tagged with the first 55 amino acids of human herpes virus glycoprotein D (HHV gD1) at the N terminus, thus all subsequent hSMO constructs were detectable using an anti-gD1 antibody. *hSMO-M2* was mutagenized to *hSMOWT* using the primers 5′-CCATGAGCACCTGGGTCTGGACCAAG and 5′-CTTGGTCCAGACCCAGGTGCTCATGG. To make *hSMOCLD*, we mutagenized two sequential amino acids, W545A and R546A, using the primers 5′-CATCGCGGCGCGTACCTGGTGCAG and 5′-GTACGCGCCGCGATGAGCAGCGTG. These amino acids were equivalent to W549A, R550A mutations in mouse *Smo*, which cause a ciliary localization defect (Bijlsma et al., 2012; Corbit et al., 2005). We confirmed *in vitro* that the *hSMO* constructs were not effectively downregulated by shSMO, which was designed against chicken *Smo* (Fig. S6D). For *in ovo* electroporations (using 50 ng/µl), the *Smo* variants were cloned into pMES, which is driven by a β-actin promoter and contains an IRES-GFP sequence to identify the transfected cells. The *Smo* variants were excised from pRK7 using HindIII (blunted) and EcoRI, whereas pMES was digested with XbaI (blunted) and EcoRI. The fragments were ligated using T4 DNA ligase (all enzymes from New England Biolabs).

### Mouse Arl13b-GFP construct

Mouse Arl13b-GFP (mArl13b-GFP) constructs were subcloned from the one used in Das and Storey (2014) with either a β-actin promoter or a Math1 enhancer and a minimal β-globin promoter (mArl13bWT-GFP variants). The V358A mutation was incorporated into both resulting plasmids using site-directed mutagenesis (mArl13bV358A-GFP variants). For the rescue experiments Math1 plasmids were injected and electroporated at a concentration of 1000 ng/µl together with 25 ng/µl of β-actin::hrGFPII plasmid at HH17-HH18. For assessing the localization of the two different mArl13b-GFP WT and V358A proteins, the plasmids with β-actin promoter were injected and electroporated at a concentration of 30 ng/µl at HH17-HH18.

### Electroporation and assessment of axon guidance phenotypes

A detailed video protocol demonstrating the electroporation, dissection and DiI injection steps in chicken embryos is available online (Wilson and Stoeckli, 2012). In brief, embryos were injected and electroporated at HH17-HH18, using a BTX ECM830 square-wave electroporator (five pulses of 25 V, 50 msec duration, 1 s interpulse interval). Targeted dorsal and ventral electroporations were achieved by careful positioning of the electrodes relative to the neural tube, and successful targeting was verified by hrGFPII expression. The resulting axon guidance phenotypes were assessed by axonal tracing with DiI in open-book preparations of spinal cords. Note that the downregulation and rescue experiments of Smo were performed at ∼HH15-HH16 to efficiently downregulate Smo. The spinal cords of E12.5 mice or HH25-HH26 chicken embryos were dissected, and ‘open-book’ preparations were made by cutting along the roof plate and pinning the spinal cord open with the basal sides down, as depicted in Fig. 2F. At least seven embryos were examined in each condition by a person unaware of the experimental condition. Fast-DiI (5 mg/ml in ethanol; Molecular Probes) was applied by focal injection into dorsal commissural neurons. Labeled axons at the midline were documented by confocal microscopy (Olympus DSU coupled to BX61 microscope). Only DiI injections sites that were in the appropriate location in the dorsal-most part of the spinal cord, and (for the chicken embryos) within the region expressing fluorescent protein, were included in the analysis. As it was impossible to count axons at individual injection sites, the percentage of axons displaying abnormalities was estimated, and the injection site was classified as showing a ‘FP stalling’ phenotype if >50% of axons stalled within the floorplate, or a ‘post-crossing’ phenotype if >50% of axons that reached the contralateral floorplate border failed to make a correct turn into the longitudinal axis. At a single abnormal DiI injection site, it was possible that more than one class of phenotypic error was observed. The total number of DiI sites in each condition was pooled and the percentage of normal injections sites were statistically compared across conditions.

### *In situ* hybridization

ISH and immunolabeling were performed as previously described (Mauti et al., 2006; Wilson and Stoeckli, 2011). All sense and antisense ISH probes were generated using SP6, T7 or T3 RNA polymerase, and DIG RNA Labeling mix (Roche). The chick *Hhip* probe has been previously described (Bourikas et al., 2005). Images were acquired using a BX63 microscope (Olympus) and a 20× air objective (ACHN P 20×/0.4, Olympus) and an Orca-R^2^ camera (Hamamatsu) with the Olympus CellSens Dimension 2.2 software.

For comparison of the *Shh* expression patterns along the longitudinal axis of the spinal cords, we embedded several fixed embryos in the same block, to enable a comparison of different embryos at the same axial level while minimizing slide-to-slide variability. The embryos were laid side-by-side in the supine position in O.C.T. compound (Tissue-Tek), and we aligned their hindlimbs and forelimbs perpendicular to the cutting surface before freezing. Then 25 µm-thick cryostat sections at 400 µm intervals were collected on slides, such that each slide contained ten sections that spanned from the hindlimb to the forelimb level. Analysis of relative *Shh* mRNA levels on the control and electroporated sides of the spinal cords was performed as described in Wilson and Stoeckli (2013).

For *Hhip in situ* quantification in dI1 neurons of the electroporated versus the non-electroporated side on cryosections (Figs 5 and 7), images were inverted and the mean value within a circular area positioned dorsally to the dorsal funiculus was measured on both control and electroporated side. Another value with the same circle was taken in the motor column of the un-electroporated (control) side. As *Hhip* is not expressed in the motor column (Wilson and Stoeckli, 2013) and as the density of cells in this area is quite similar to the area with the dI1 neurons, we used it as a measure of background. This background value was subtracted from each *Hhip* mean value in dI1 neurons and the ratio electroporated:control side was then calculated for each section. A minimum of 10 sections were quantified per embryo and the average ratio for each condition was normalized to the average ratio of GFP controls (Fig. 5A-C) or hSmoWT rescue (Fig. 7A-D). For comprehensive representation *in situ* images were inverted and Royal LUT implemented in ImageJ to highlight accumulation or reduction of mRNA in dI1 neurons (Figs 5B,C and 7B-D).

### Immunohistochemistry for neuronal patterning

Mouse or chicken embryos were killed, dissected and fixed in 4% PFA in PBS for 1 h at room temperature, as described. After being washed three times for 5 min each in PBS, they were cryopreserved for 24 h in 25% sucrose in PBS at 4°C and then embedded in O.C.T. compound. From the trunk, 25 µm thick transverse sections were cut, permeabilized for 10 min at room temperature with 0.1% Triton X-100 in PBS and blocked for 1 h in 0.1% Triton X100, 5% fetal calf serum (FCS) in PBS (blocking buffer). Cryosections were incubated overnight at 4°C in the following primary antibodies diluted in blocking buffer: mouse anti-Pax3 [supernatant, Developmental Studies Hybridoma Bank (DSHB), PAX3; RRID: AB_528426], mouse anti-Islet1 (supernatant, DSHB, 40.2D6; RRID: AB_528315), mouse anti-Shh (supernatant, DSHB, 5E1; RRID: AB_528466), mouse anti-FoxA2(HNF3β) (supernatant, DSHB, 4C7; RRID: AB_528255), mouse anti-Lhx2 (supernatant, DSHB, PCRP-LHX2-1C11; RRID: AB_2618817), mouse anti-Nkx2.2 (supernatant, DSHB, 74.5A5; RRID: AB_531794), goat anti-GFP-FITC (1:400, Rockland, 600-102-215; RRID: AB_218187), goat anti-Robo3 (1:500, R&D Systems, AF3076; RRID:AB_2181865) or rabbit anti-Axonin-1 (1:1000; Stoeckli and Landmesser, 1995). The next day, sections were washed three times for 10 min each in 0.1% Triton X-100 in PBS at room temperature and then incubated for 2 h with secondary antibodies diluted in blocking buffer: donkey anti-rabbit-IgG-Cy3 antibody (1:1000, Jackson ImmunoResearch, 711-165-152; RRID: AB_2307443), donkey anti-mouse-IgG-Cy5 antibody (1:1000, Jackson ImmunoResearch, 715-175-150; RRID: AB_2340819) or donkey anti-mouse-IgG-Cy3 (1:1000, Jackson ImmunoResearch, 715-165-150; RRID: AB_2340813). Finally, sections were washed three times for 10 min  each in 0.1% Triton X-100 in PBS and twice for 5 min each in PBS before being mounted under a coverslip in Mowiol/DABCO. Images were acquired with a BX63 upright microscope (Olympus) and a 20× air objective (ACHN P 20×/0.4, Olympus) and an Orca-R^2^ camera (Hamamatsu) with the Olympus CellSens Dimension 2.2 software.

### Immunohistochemistry of primary cilia

For staining of neuronal cilia in the neural tube, we had to modify certain steps of the protocol to increase signal to background of ciliary staining (Yusifov et al., 2021). Chicken embryos were sacrificed and dissected as described above in warm (37°C) PBS and then fixed at room temperature in pre-warmed 4% PFA in PBS for different times according to the stage (HH20, 35 min; HH22, 40 min; HH24, 40 min; HH26, 45 min). Note that this initial change in the protocol was not required for mouse embryos. Embedding, sectioning and immunostaining for Lhx2 and GFP were performed as detailed above. Once this staining was finished and sections washed with PBS, they were incubated for 2 h in primary antibodies diluted in 5% FCS in PBS at room temperature to stain primary cilia with either rabbit anti-Arl13b (1:500, Proteintech, 17711-1-AP; RRID: AB_2060867), rabbit anti-Ift88 (1:1000, Proteintech, 13967-1-AP; RRID: AB_2121979) or rabbit anti-Adenylyl cyclase III (ACIII, 1:500, Santa Cruz Biotechnology, sc-588; RRID: AB_630839). Sections were then washed three times for 10 min each in PBS and stained for 2 h with donkey anti-rabbit-IgG-Cy3 antibody (1:1000, Jackson ImmunoResearch, 711-165-152; RRID: AB_2307443) and 2.5 μg/ml of Hoechst (H3570, Invitrogen) diluted in PBS. Finally, they were washed three times for 10 min each in PBS and mounted as detailed above. Images of cilia stainings were acquired using a BX61 upright microscope (Olympus) equipped with a spinning disk unit either with a 10× air objective (overviews; UPLFL PH 1×/0.30, Olympus) or a 60× oil objective (PLAPON O 60×/1.42, Olympus) and an Orca-R^2^ camera (Hamamatsu) with the Olympus CellSens Dimension 2.2 software, or with an IX83 inverted microscope equipped with a spinning disk unit (CSU-X1 10,000 rpm, Yokogawa) with a 60× silicone oil objective (UPLSAPO S2 60×/1.30) and an Orca-Flash 4.0 camera (Hamamatsu) with the Olympus CellSens Dimension 2.2 software.

Quantification of ciliation in WT HH24 spinal cord was carried out in Fiji as described previously (Yusifov et al., 2021) for dorsal root ganglia. In brief, the Lhx2-positive region containing the dI1 somas was selected as a region of interest (ROI). The ROI was then transferred to the Arl13b channel and a threshold (renyEntropy) was applied to get rid of the background/extraciliary staining. Finally, the remaining particles/dots were automatically counted using the ‘analyze particles’ feature in Fiji giving the number of cilia within the ROI which was then divided by the number of Lhx2-positive nuclei in the ROI to get the percentage of dI1 neuron ciliation.

### Live imaging of intact spinal cords

Live imaging of intact chicken spinal cords was performed as previously described (Dumoulin et al., 2021). In brief, the neural tube of HH17-HH18 embryos was injected and unilaterally electroporated *in ovo* as described above (25 Volts, five pulses of 50 ms length with 1 s interval between pulses) with 700 ng/µl Math1::tdTomato-F plasmid to label dI1 neurons and 30 ng/µl of β-actin::EGFP-F plasmid as control in all transfected cells (Dumoulin et al., 2021). Intact spinal cords were dissected at HH22 and cultured in a 100-µl drop of low-melting agarose, with the ventral midline facing down, on a 35 mm Ibidi μ-dish with glass bottom (Ibidi, 81158) and spinal cord medium [MEM with Glutamax (Gibco) supplemented with 4 mg/ml Albumax (Gibco)], 1 mM pyruvate (Sigma-Aldrich), 100 units/ml Penicillin and 100 μg/ml Streptomycin (Gibco), as previously described (Dumoulin et al., 2021).

Intact spinal cords were cultured at 37°C, with 5% CO_2_ and 95% air in a PeCon cell vivo chamber (PeCon). CO_2_ percentage and temperature were controlled by the cell vivo CO_2_ controller and the temperature controller units, respectively (PeCon). Spinal cords were incubated for 30 min in the chamber before the live imaging acquisition was initiated using an IX83 inverted microscope equipped with a spinning disk unit (CSU-X1 10,000 rpm, Yokogawa) and a 20× air objective (UPLSAPO 20×/0.75, Olympus) and an Orca-Flash 4.0 camera (Hamamatsu) with the Olympus CellSens Dimension 2.2 software. We acquired one stack of 30-45 slices with 1.5 µm spacing every 15 min for 24 h with 488 nm and 561 nm emission channels, as well as the bright-field channel to visualize the structure of the midline area. For the Smo inhibition experiments, either a final concentration of 15 µM Cyclopamine diluted in ethanol (MedChemExpress, HY-17024) or of 20 µM Itraconazole diluted in DMSO (Sigma-Aldrich, I6657) was added to the spinal cord medium. Control conditions consisted of spinal cords cultured in 1:1000 dilution of either ethanol (Cyclopamine control) or DMSO (Itraconazole control). As no obvious defects in midline crossing were detected upon Smo inhibition, we focused our quantification of aberrant phenotypes on the post-crossing segment of Math1-positive dI1 axons. An axon was considered to have an aberrant phenotype if it stalled or turned caudally instead of rostrally at the contra-lateral border of the floorplate. Tracing/counting of dI1 axons, processing of time-lapse video and video montages were performed using Fiji/ImageJ (Schindelin et al., 2012).

### Commissural neuron cultures in microfluidic chambers

The most dorsal part of the spinal cord, containing mostly dI1 commissural neurons, was cut from 6-7 open-book preparations of WT HH25-HH26 embryos in ice-cold sterile PBS as previously described (Yusifov et al., 2021). Cells were dissociated with 0.25% Trypsin (Invitrogen, 15090-046) in PBS, containing 0.2% DNase (Roche, 101 041 590 01), at 37°C for 20 min followed by a sequence of trituration in different pre-warmed media [37°C; MEM (Gibco) with 5% FCS (Gibco), MEM only, and finally in commissural neuron medium] with fire-polished Pasteur pipettes and centrifugation at 1000 rpm (175 ***g***) for 5 min at room temperature in-between. Commissural neuron medium contained MEM/Glutamax (Gibco, 41090-028) supplemented with 4 mg/ml Albumax (Gibco), N3 (100 µg/ml transferrin, 10 µg/ml insulin, 20 ng/ml triiodothyronine, 40 nM progesterone, 200 ng/ml corticosterone, 200 µM putrescine, 60 nM sodium selenite; all from Sigma-Aldrich) and 1 mM pyruvate (Sigma-Aldrich). Dissociated cells (130,000) in 20 µl commissural neuron medium were added to the upper left well of Xona chip 150 µm microfluidic chambers connected to the soma chamber (Xona, XC150). After 5 min at room temperature, 100 µl of medium were given to all wells. Plates were incubated at 37°C with 5% CO_2_ for 1 or 8 days. Every 2 days, 50% of the volume was changed in each well with freshly prepared medium. The microfluidic chips were coated with 20 µg/ml poly-L-lysine (Sigma-Aldrich, P-12374) and 20 µg/ml laminin (Invitrogen, 23017-015) following the protocol of the manufacturer (XonaChip^TM^ protocol for primary murine neurons). On the day of stimulation (1 or 8 days *in vitro*), culture medium was discarded from all wells, and 140 µl commissural neuron medium were added to both left wells (connected to the soma chamber) and 130 µl of the same medium containing 2.5 µg/ml recombinant ShhN-6xHis [R&D Systems, 1845-SH; 1:40 dilution in medium of stock solution solubilized in 0.1% bovine serum albumin (BSA)] were given to both wells connected to the axonal chamber. Note that, in control experiments without recombinant Shh (Fig. 8G-I), the equivalent amount of 0.1% BSA (1:40 dilution in medium) was given to the well connected to the axonal chamber. Cells were then incubated at 37°C with 5% CO_2_ for 7 h. Afterwards, the medium from the right side (axonal chamber) was discarded before the one in the soma chamber and then cells were washed once with pre-warmed (37°C) commissural neuron medium, first adding 100 µl in the left wells and 80 µl in the right wells. Then, cells were fixed with pre-warmed (37°C) 2% PFA in PBS and incubated at 37°C with 5% CO_2_ for 5 min before 15 min under the same conditions in 4% pre-warmed PFA in PBS. Finally, cells were washed three times for 5 min each with PBS (150 µl per well) at room temperature and stored at 4°C until immunocytochemistry was performed.

For the co-culture of HEK cells expressing Shh-mRuby3, HEK cells were transfected with the β-actin::Shh-mRuby3 plasmid using polyethylenimine (PEI) 3 days before the experiment. Cells were detached and 120,000 cells were given to the axonal compartment of 8 days *in vitro* (DIV) cultures of commissural neurons in one well connected to the axonal chamber and the culture medium was added to reach a total volume of 260 µl in the axonal compartment versus 280 µl in the compartment containing the neuronal somas. Note that the density of HEK cells was very low as they did not adhere well. However, most of the cells that remained in the chamber were in contact with axons. After overnight co-culture, single-plane live snapshots were acquired at the soma level of commissural neurons with a spinning disk microscope using the same live imaging setup described above with a 40× silicone oil objective (UPLSAPO S 40×/1.25, Olympus), and an Orca-Flash 4.0 camera (Hamamatsu) with the Olympus CellSens Dimension 2.2 software.

### Immunocytochemistry microfluidic chambers

Importantly, for successful staining in microgrooves of the microfluidic chamber, the addition of any buffer in the wells was always 40 µl higher on one side of the dish (typically 150 µl in each of the two left wells and 130 µl in each of the two right wells). Neurons were first permeabilized with 0.1% Triton X-100 in PBS for 5 min at room temperature and washed three times for 5 min each with PBS. They were then blocked for 15 min in 5% FCS in PBS (blocking buffer). Samples were stained overnight with the following primary antibodies diluted in blocking buffer: mouse anti-neurofilament-M (1:1500, RMO270, Invitrogen; RRID: AB_2315286); rabbit anti-6xHisTag (1:2000, Rockland, 600-401-382) and goat anti-Axonin-1 (1:1000; Stoeckli and Landmesser, 1995). For Arl13b staining, samples were incubated for 1 h at room temperature with the primary antibody (rabbit anti-Arl13b, 1:1000, ProteinTech, 13967-1-AP; RRID:AB_2121979). After being washed three times for 5 min each with PBS at room temperature, neurons were stained for 2 h (1 h for Arl13b staining) at room temperature with secondary antibodies (same as used for immunohistochemistry) diluted in blocking buffer. At the end, they were counterstained for 5 min with Hoechst (2.5 µg/ml, Invitrogen, H3570) diluted in PBS at room temperature and washed three times for 5 min each with PBS.

Images were acquired using either an IX81 inverted microscope (Olympus), with a 10× air objective (UPLFL PH 10×/0.30, Olympus) or a 60× oil objective (PLAPO O 60×/1.40, Olympus, Fig. 8A,B), or with an IX83 inverted microscope, equipped with a spinning disk unit (CSU-X1 10,000 rpm, Yokogawa) and a 40× silicone oil objective (UPLSAPO S 40×/1.25, Olympus) or a 60× silicone oil objective (UPLSAPO S2 60×/1.3, Olympus), and an Orca-Flash 4.0 camera (Hamamatsu) with the Olympus CellSens Dimension 2.2 software. The same acquisition settings were used to take pictures of the 6xHisTag staining (recombinant Shh) in all conditions. Three independent experiments were performed with similar outcomes.

### Live imaging of explant co-cultures

For dorsal spinal cord explants, spinal cords were unilaterally electroporated with 700 ng/µl of dI1 neuron-specific Math1::tdTomato-F plasmid at HH17-HH18 as described above. For floorplate explants, the floorplate-specific Hoxa1::EGFP-F plasmid (1000 ng/µl; Dumoulin et al., 2021) together with β-actin::Shh-mRuby3 (200 ng/µl) were electroporated ventrally with the same voltage and regime as above. The mRuby3 sequence was subcloned from the pKanCMV-mRuby3-18aa-actin plasmid that was obtained from Michael Lin (Addgene plasmid #74255, RRID: Addgene_74255) into the chicken Shh full-length sequence (Bourikas et al., 2005) using the NEBuilder^®^ HiFi DNA Assembly kit (New England Biolabs) (Fig. 8M). The mRuby3 sequence was incorporated after the Glycine200, and the sequence Alanine190-AENSVAAKSG-Glycine199 was duplicated and incorporated downstream of the mRuby3 sequence as previously described with mouse Shh (Hall et al., 2021). This allowed Shh to retain its biological activity (Chamberlain et al., 2008; Hall et al., 2021) (Fig. 9A-C).

Dorsal spinal cord explants and floorplate explants were dissected from open-book preparations 1 or 2 day(s) after electroporation at HH22 and HH25-HH26, respectively. Explants were plated on eight-well live-imaging plates (µ-Slide, IBIDI, 80826) coated with 20 µg/ml poly-L-lysine (Sigma-Aldrich, P-12374) and 20 µg/ml laminin (Invitrogen, 23017-015). Dorsal spinal cord explants were plated with the apical side facing the glass surface and the ventral side facing the floorplate explants. Floorplate explants were either positioned in close proximity so that they were in contact with the dorsal spinal cord explants and formed a continuous 3D structure after a few hours in culture or at a distance (at least 100 µm). Explants were cultured in the same set-up as used for intact spinal cords with controlled temperature and CO_2_ settings (see above).

Explant co-cultures (fused) were imaged with a 20× air objective (UPLSAPO 20×/0.75, Olympus) and an Orca-Flash 4.0 camera (Hamamatsu) with the Olympus CellSens Dimension 2.2 software. One stack of 17 slices with 1.5 µm spacing between slices was taken every 5 min for 2.5 h. Co-cultures of explants put at a distance were imaged with the same microscope but using the widefield mode with a 100× silicone oil objective (UPLSAPO S 100×/1.35, Olympus) and an Orca-Flash 4.0 camera (Hamamatsu) with the Olympus CellSens Dimension 2.2 software. One image was taken every second for a maximum of 1 min because of bleaching of the Shh-mRuby3 signal. Maximum projections and single planes of time-lapse recording were corrected for bleaching, processed and videos were made in Fiji/ImageJ. The kymographic analysis was performed using the re-slice function and the *z*-projection of the re-sliced results was made in Fiji/ImageJ.

### Visualization of Shh *in vivo*

Visualization of endogenous or mRuby3-tagged Shh was carried out on 300 µm-thick tissue chopper slices of trunks from HH23.5 embryos that were previously fixed for 30 min with 4% PFA in PBS at room temperature. Shh is known to be secreted in very small nanoparticles (lipoprotein particles). Such particles might be destroyed by the use of a detergent. Thus, we developed a staining protocol for trunk slices in which we avoided the use of a detergent and were able to see endogenous Shh using the 5E1 antibody. Slices were first blocked for 30 min at room temperature with 5% FCS in PBS. Then, they were incubated at 4°C for 3 days with mouse anti-Shh (5E1, RRID: AB_2188307) together with rabbit anti-Axonin-1 antibodies diluted in 5% FCS in PBS and washed three times for 15 min each with PBS. Then, slices were incubated in secondary antibodies [donkey anti-mouse-IgG-Cy3 (1:1000, Jackson ImmunoResearch, 715-165-150; RRID: AB_2340813) and donkey anti-rabbit_IgG-488 (1:1000, Invitrogen, A-21206; RRID: AB_2535792)] diluted in 5% FCS in PBS overnight at 4°C, before being washed three times for 15 min each at room temperature. Slices were mounted in a drop of PBS between two coverslips, positioned in the center of a squared border made of vacuum grease. Imaging was performed using an IX83 inverted microscope equipped with a spinning disk unit (CSU-X1, 10,000 rpm, Yokogawa) and an Orca-Flash 4.0 camera (Hamamatsu). The microscope was operated with the Olympus CellSens Dimension 2.2 software. Depending on the specific experiment, we used a 40× silicone oil objective (UPLSAPO S 40×/1.25, Olympus) (Fig. 9J), a 60× silicone oil objective (UPLSAPO S2 60×/1.3, Olympus) (Fig. 9L) or a 20× air objective (ACHN P 20×/0.4, Olympus) (Fig. 9N). The Shh-mRuby3 construct was subcloned under the Hoxa1 enhancer and the β-globin minimal promoter to drive floorplate-specific expression (Wilson and Stoeckli, 2011). This plasmid was ventrally electroporated (10 µg/µl) at HH18-HH19 as described above. Immediately after fixation, samples were sliced, mounted and imaged as described above to detect endogenous fluorescence of Shh-mRuby3 in intact spinal cords.

### Statistical analyses

Statistical analyses were performed with the GraphPad Prism 7.02 software. All data were tested for normality (normal distribution) using the D'Agostino and Pearson omnibus K2 normality test and visual assessment of the normal quantile-quantile plot before choosing an appropriate (parametric or non-parametric) statistical test. For data and statistics, see Table S1.

## Supplementary Material

10.1242/develop.202788_sup1 Supplementary information

Table S1. Source data
