# Supplementary material for: A cell-autonomous role for primary cilium-mediated signaling in long-range commissural axon guidance
Source: Development. 2024 Sep 5;151(17):dev202788. doi: 10.1242/dev.202788 (PMC11423920; doi:10.1242/dev.202788)
Supplement: Supplementary information [file develop-151-202788-s1.pdf]

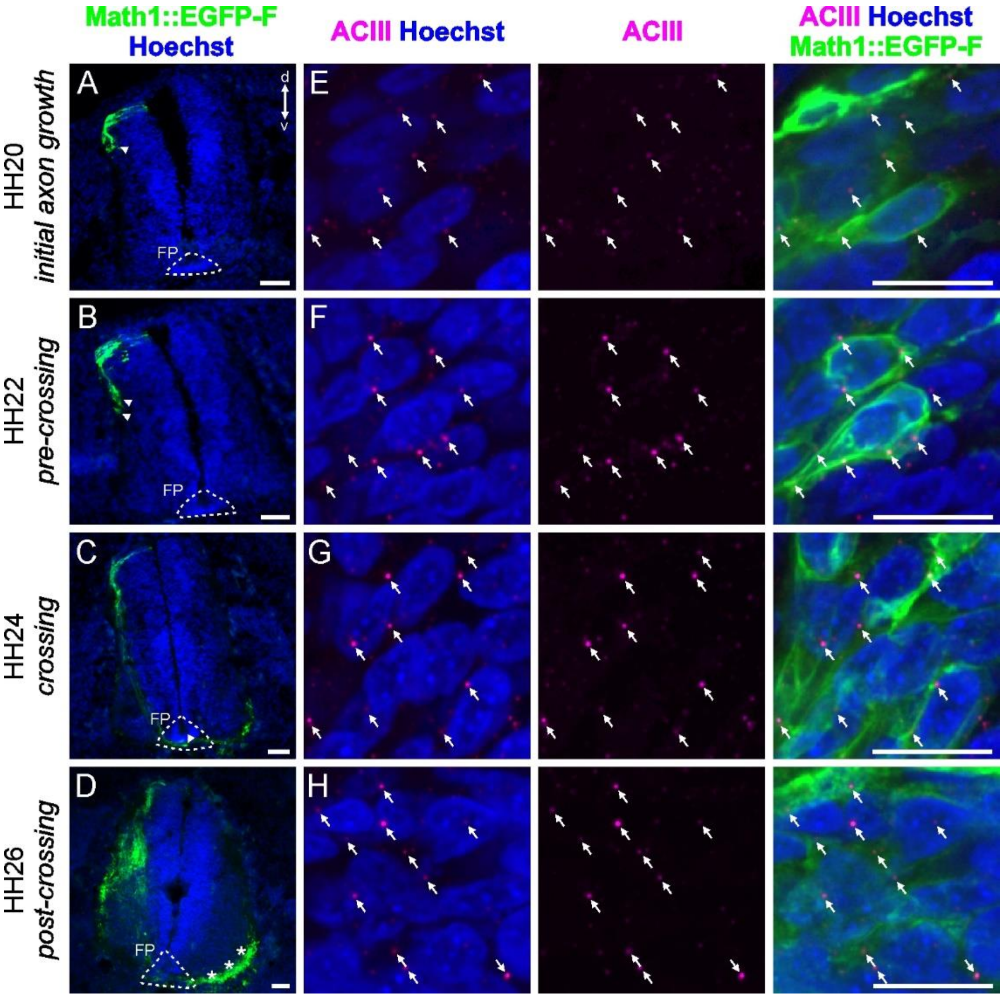

Fig. S1. dI1 commissural neurons bear a primary cilium at different time points of their development *in vivo*.

(A-D) Transverse sections of chicken embryos sacrificed at different time points of axonal navigation. The dI1 subpopulation of commissural neurons were labeled by unilateral electroporation of Math1::EGFP-F plasmid (green) at HH17-18. At HH20, dI1 axons started to extend (A), at HH22 they were growing ventrally (B), at HH24 they were crossing the floorplate (C) and at HH26 they were localized in the post-crossing segment (D). White arrowheads show where dI1 axonal growth cones localized at the different time points. Asterisks in (D) label axons extending in the ventral funiculus. Sections were counterstained with Hoechst to stain nuclei (blue). (E-H) High magnification images of the area of dI1 neurons in the dorsal spinal

Development • Supplementary information

cords depicted in (A-D) showing Math1-positive dI1 neuron somas (expressing EGFP-F) co-stained with the primary cilium marker ACIII (magenta). These neurons carried a primary cilium (white arrows) at their soma level throughout stages HH20 to HH26. FP, floorplate; d, dorsal; v, ventral. Scale bars: 50 µm (A-D) and 10 µm (E-H).

Development • Supplementary information

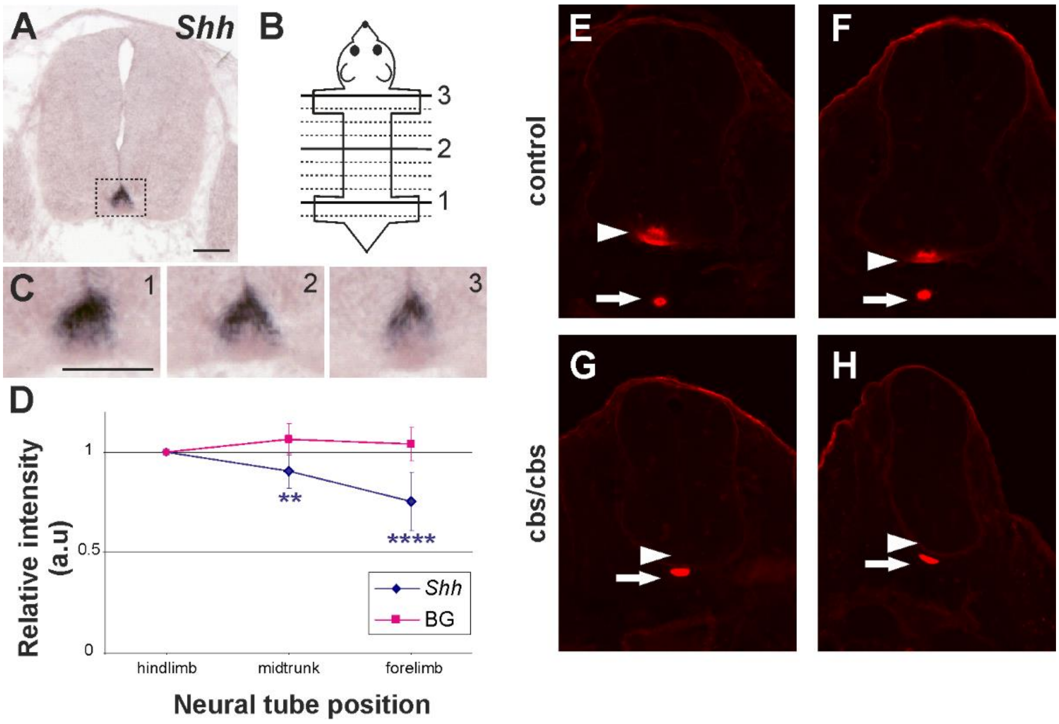

Fig. S2. Shh is expressed in an anterior-posterior gradient in the mouse spinal cord.

(A) In situ hybridization for Shh shows specific expression in the floorplate (boxed area), in a transverse section of mouse E12.5 spinal cord. (B) Schematic illustration of the method used to collect sections spanning the longitudinal axis. Each slide contained 10 sections at 400 µm intervals. (C) Representative images of Shh in the floorplate at axial levels corresponding to hindlimb (1), midtrunk (2), and forelimb (3) of the same embryo. (D) Plot of relative Shh intensity versus relative position along the neural tube (mean±SEM; n=12 embryos; single-sample T-test; $p<0.0001$ (****), $p<0.01$ (**)). Background (BG) staining levels, sampled from an area in the dorsal spinal cord, did not change along the A-P axis. a.u., arbitrary units. In wildtype embryos (+/+), Shh protein was also found in a decreasing gradient with higher levels in the caudal (arrowhead, E) and lower levels in the rostral floorplate (arrowhead, F). No or only very low levels of Shh were found in the floorplate of cbs/cbs mice (arrowhead, G,H). Shh was still expressed in the notochord of cbs/cbs mice (arrows). Scale bars: 100 µm. Source data and statistics are available in Table S1.

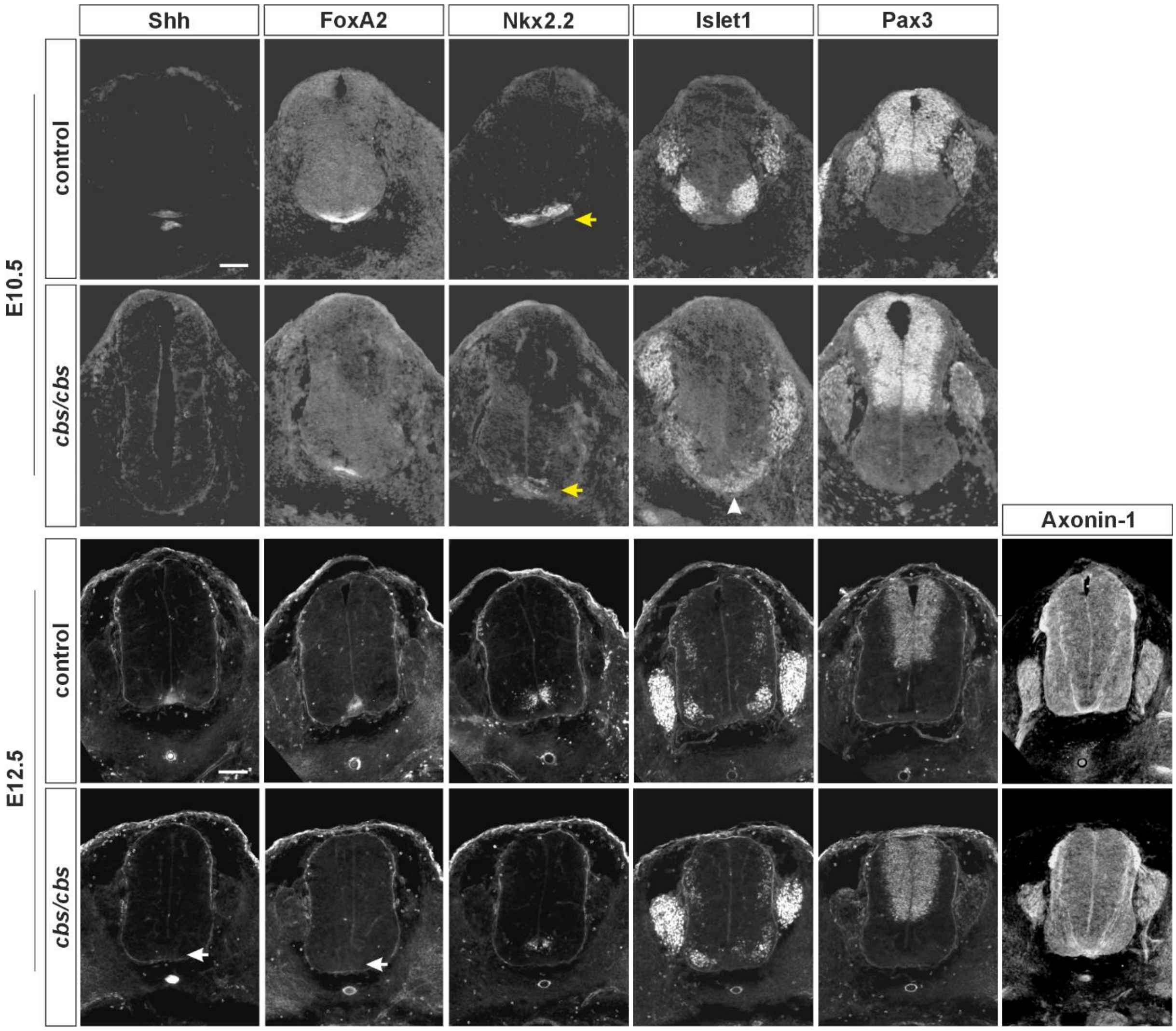

Fig. S3. Patterning defects are seen in the ventral but not the dorsal spinal cord of cbs/cbs mice. Immunostaining for an array of spinal cord markers (as indicated) at E10.5 and E12.5 revealed several defects in cbs/cbs embryos (arrows). Shh and the floorplate marker FoxA2/Hnf3β were both missing from cbs/cbs spinal cords at E10.5 and E12.5. The ventral marker Nkx2.2 was present at both stages, but was reduced and disorganized, especially at E10. Islet1-positive cells erroneously invaded the ventral midline of E10.5 cbs/cbs mice, but by E12.5, Islet1 expression resembled that in control littermates. In contrast, the dorsal marker Pax3 was expressed normally in cbs/cbs mice. Dorsal commissural neurons, labeled by Axonin1/ Contactin2, were also found in their normal position, and projected correctly to the ventral spinal cord. However, the ventral commissure appeared defasciculated in cbs/cbs mice, as expected from our analysis of axon projections at the midline in open-book preparations. Scale bars: 50 µm.

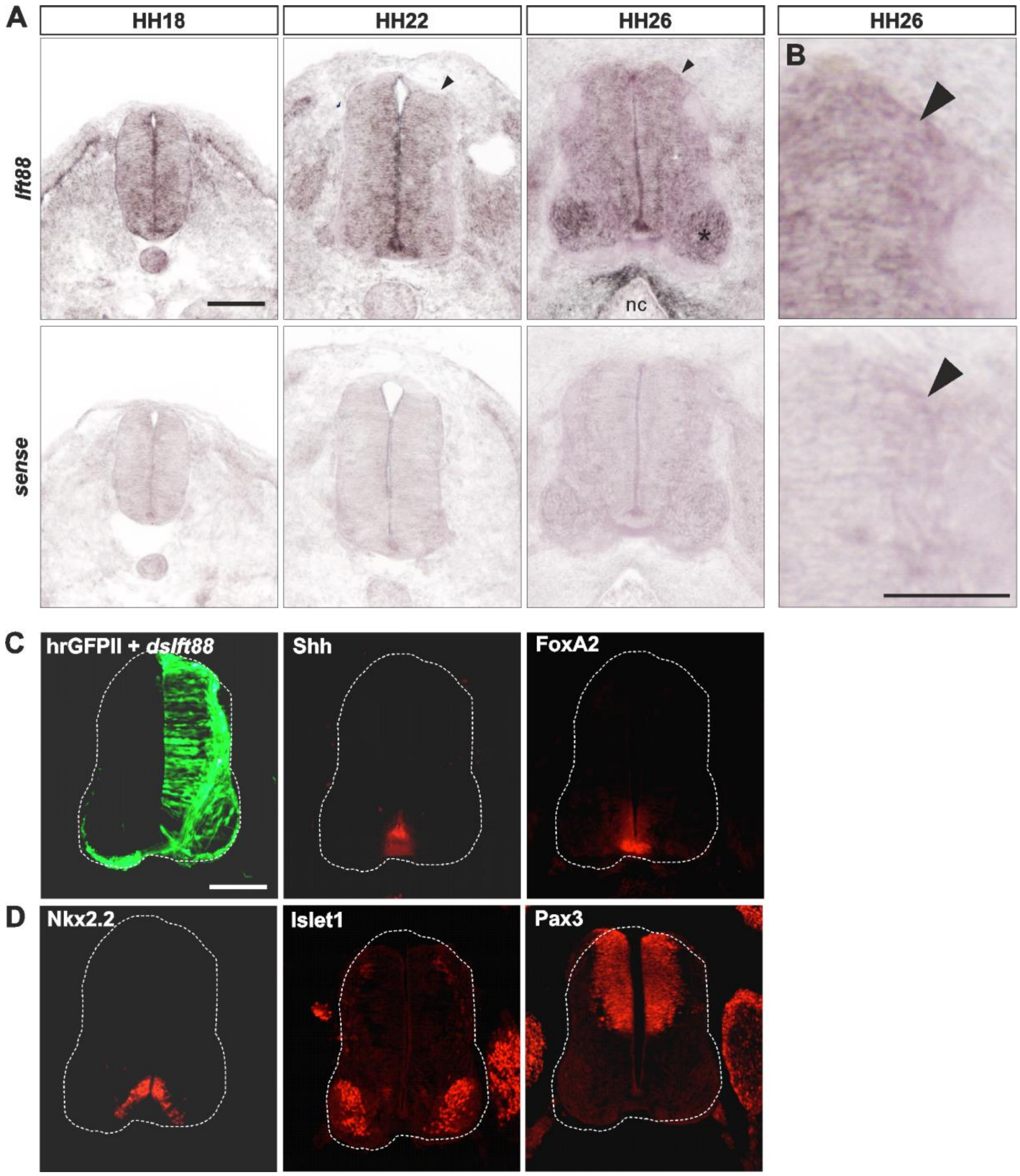

Fig. S4. Ift88 is expressed ubiquitously in the chicken spinal cord, but its knockdown after HH18 does not affect spinal cord patterning.

(A) In situ hybridization for *Ift88* at the indicated developmental stages (top) shows expression throughout the embryonic chicken spinal cord, including the area occupied by the commissural neurons (arrowheads). A higher magnification of the area of dI1 neurons is shown in (B) for HH26. At HH18 and HH22, levels in precursors (ventricular zone) appeared to be higher than the expression in mature neurons. At HH26, *Ift88* is expressed at slightly higher

Development • Supplementary information

levels in the motoneurons (asterisk) but levels were no longer higher in the ventricular zone compared to the mantle zone. At this stage, *Ift88* mRNA was also found surrounding the notochord (nc). No specific signal was seen with a sense control probe (second row). (C,D) Immunostaining for a panel of spinal cord markers (as indicated) reveals normal patterning after electroporation of dsIft88 at HH18. The electroporated side was identified by expression of hrGFPII (green) from a co-electroporated plasmid. Downregulation of Ift88 at HH18, after spinal cord patterning is completed, did not affect Shh or FoxA2/HNF3β expression in the floorplate (C), nor affect patterning, as no difference in the expression of Nkx2.2, Islet1 or Pax3 were seen between the control and electroporated side (D). Scale bar 100 µm.

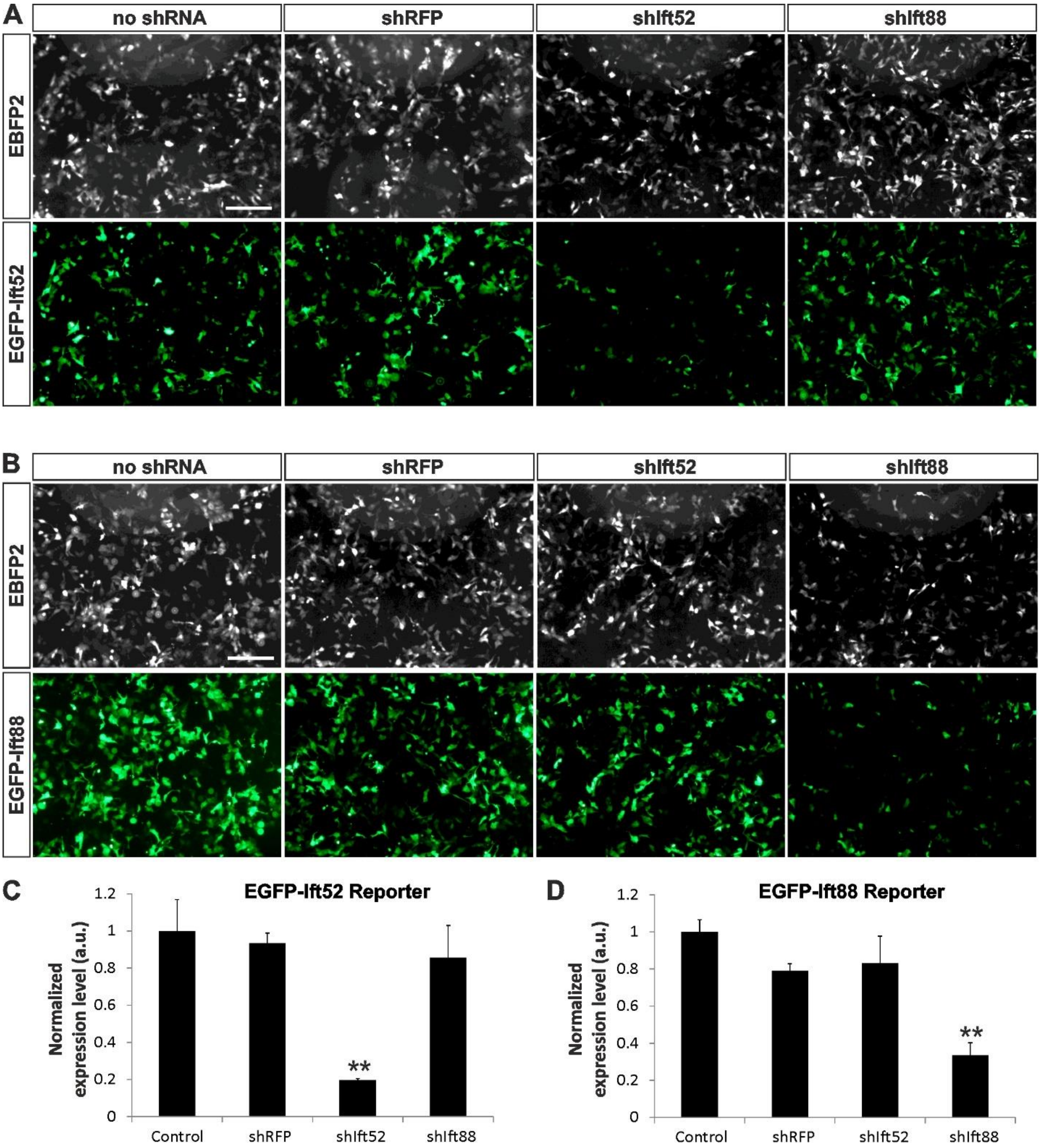

Fig. S5. Double-stranded RNAs derived from *Ift88* and *Ift52* knock down target gene sequences efficiently and specifically.

(A) COS7 cells were co-transfected with EBFP2 (top, transfection control), a reporter construct in which the *Ift52* EST sequence was cloned downstream of *EGFP* (EGFP-Ift52; bottom) and shRNAs generated from long dsRNA, as indicated. EGFP expression was used to assess the efficiency and specificity of shRNAs derived from the *Ift52* sequence. (B) COS7 cells were co-transfected as in panel (A), except the reporter construct contained an *Ift88* EST sequence cloned downstream of *EGFP* (EGFP-Ift88). (C-D) Quantifications. EGFP levels in each condition

Development • Supplementary information

were normalized to EBFP2 transfection controls, and expression levels in the control (no shRNA) conditions were set to 1.0. (C) The EGFP-Ift52 reporter was reduced by 80.5±1.0% when co-transfected with shIft52 compared to the control condition. Co-transfection of shRFP or shIft88 did not significantly affect EGFP-Ift52 reporter levels. (D) The EGFP-Ift88 reporter was reduced by 66.7±6.9% when co-transfected with shIft88 compared to the control condition. Co-transfection of shRFP or shIft52 did not significantly affect EGFP-Ift88 reporter levels. n=10 measurements each; **p<0.001; One-way ANOVA with Tukey's multiple comparisons test. Scale bar 100 µm. Source data and statistics are available in Table S1.

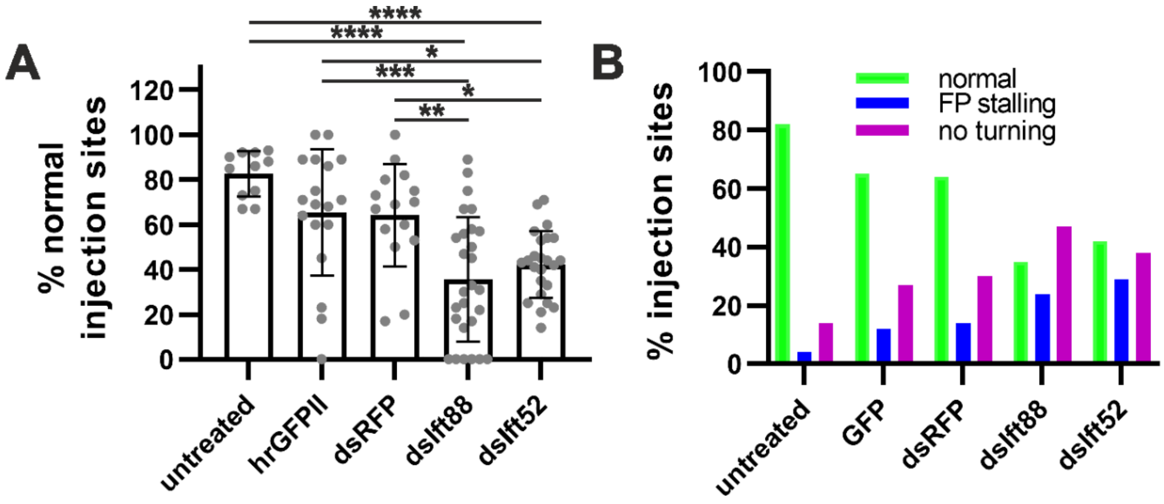

Fig. S6. The IFTB proteins Ift88 and Ift52 are required for commissural axon guidance. As expected, downregulation of Ift88 and Ift52 resulted in similar phenotypes, as they both are components of the IFTB complex. Quantification of DiI injections sites with normal axonal trajectories (A). Compared to control-treated embryos, electroporated with either a GFP-encoding plasmid or dsRNA derived from the RFP sequence, embryos electroporated with dsIft88 or dsIft52 had a significantly reduced number of DiI injection sites with normal axonal trajectories. Interference with the IFTB complex resulted in more DiI injection sites with axons stalling in the floorplate or failing to turn at the floorplate exit site (B). One-way ANOVA with Tukey's multiple comparisons test. N(embryos)=11 (untreated), 18 (hrGFPII), 15 (dsRFP), 27 (dsIFT88), 24 (dsIft52); n(injection sites)=135 (untreated), 178 (hrGFPII), 141 (dsRFP), 251 (dsIFT88), 323 (dsIft52). $p<0.0001$ (****), $p<0.001$ (***), $p<0.01$ (**), $p<0.05$ (*). For data and statistics see Table S1.

Development • Supplementary information

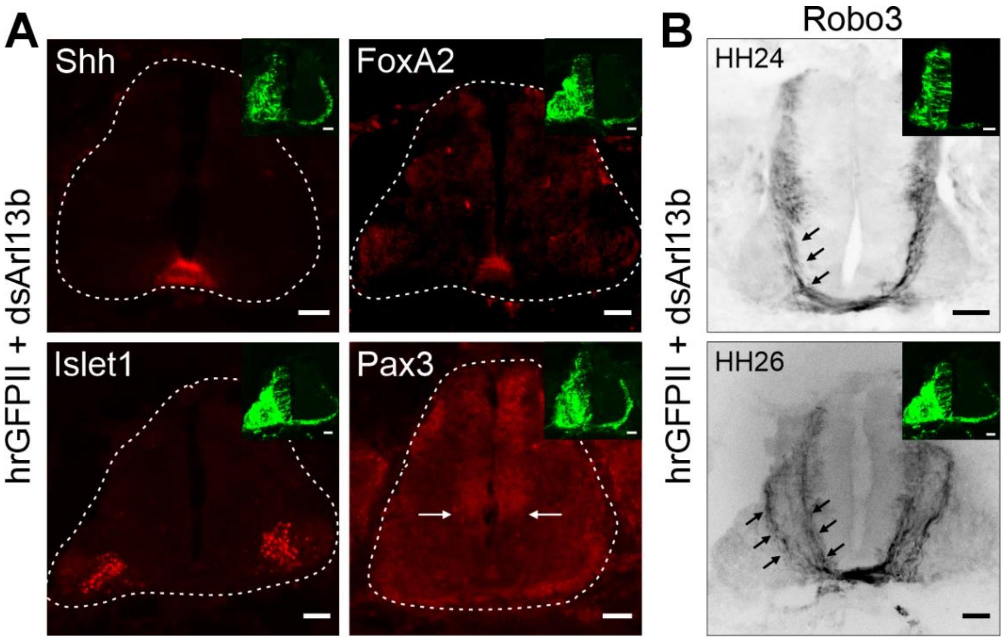

Fig. S7. Silencing Arl13b at HH17-18 did neither induce patterning defects nor affect pre-crossing commissural axon guidance.

(A) Immunostaining of cryosections of HH26 spinal cords for a panel of differentiation markers (as indicated) reveals normal patterning after electroporation of Arl13b dsRNA at HH17-18. The electroporated side was identified by expression of hrGFPII (green) from a co-electroporated plasmid (inset in the upper right corner). Downregulation of Arl13b after spinal cord patterning is completed, did not affect Shh or FoxA2/Hnf3β expression in the floorplate, nor affect patterning, as no difference in the expression of Islet1 or Pax3 were seen between the control and electroporated side. (B) Robo3 staining at HH24 and HH26 did not show any guidance defects in pre-crossing axons (black arrows). Scale bars: 50 µm.

Development • Supplementary information

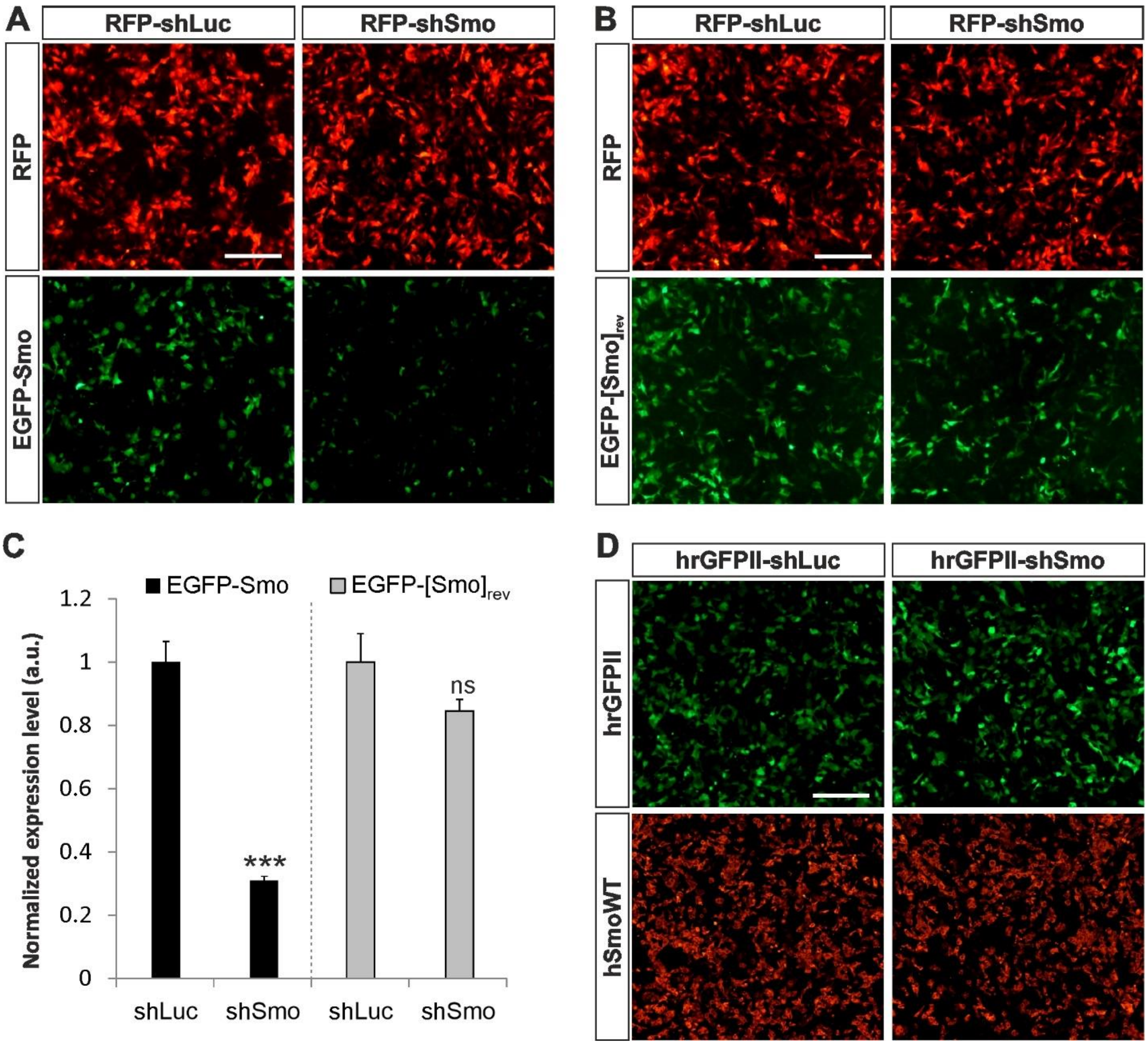

Fig. S8. shRNAs against Smoothened (shSmo) specifically and efficiently reduce target protein levels.

(A) COS7 cells were co-transfected with pRFPRNAi vectors expressing shRNAs against Luciferase (RFP-shLuc) or Smoothened (RFP-shSmo), together with a reporter construct in which a 2.2 kb fragment of chick Smo was cloned downstream of EGFP (EGFP-Smo). RFP expression (top) provided a transfection control, while EGFP expression (bottom) revealed the ability of the different shRNAs to knock down the reporter gene. (B) COS7 cells were co-transfected as in panel A, except that the reporter construct contained a 2.2 kb fragment of chick Smo that was cloned in the reverse (antisense) orientation (EGFP-[Smo]$_{rev}$). (C) Quantifications. EGFP levels in each condition were normalized to RFP levels, and expression levels in the shLuc condition were set to 1.0. The EGFP-Smo reporter was reduced by 69.1±1.4% when co-transfected with shSmo compared to shLuc. In contrast, the EGFP-

Development • Supplementary information

$[Smo]_{rev}$ reporter level was not significantly affected by shSmo transfection. n=15 measurements each; ***p<0.0001; Student's t-test. (D) COS7 cells were co-transfected with vectors expressing hrGFPII (green) and shRNAs against Luciferase (hrGFPII-shLuc) or chick Smoothened (hrGFPII-shSmo), together with a construct encoding human Smoothened (hSmoWT). Immunostaining for hSmoWT 24 hours after transfection (red) revealed that shSmo had no effect on hSmoWT expression levels compared to shLuc. Scale bars: 100 µm. Source data and statistics are available in Table S1.

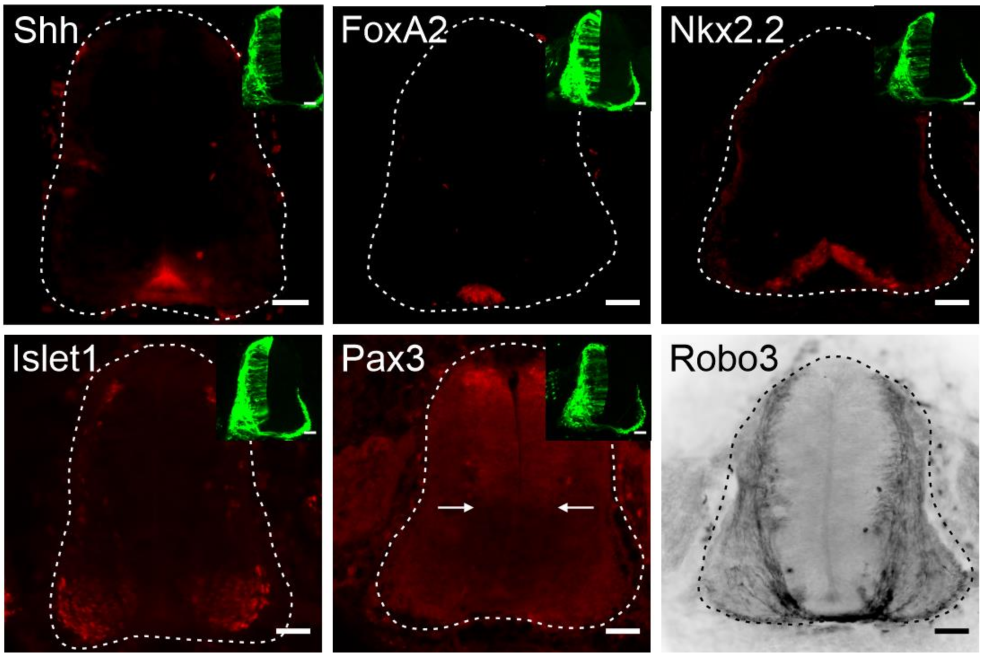

Fig. S9. Silencing Smo *in vivo* with an shRNA at HH15-16 did not induce patterning defects of the spinal cord.

(A) Immunostaining on cryosections of HH26 spinal cord for a panel of spinal cord markers (as indicated) reveals normal patterning after electroporation of shSmo at HH15-16. The electroporated side was identified by expression of hrGFPII (green) from a co-electroporated plasmid (inset in the upper right corner). Downregulation of shSmo, did not affect Shh or FoxA2/Hnf3β expression in the floorplate, nor affect patterning, as no difference in the expression of Islet1, Nkx2.2 or Pax3 were seen between the control and electroporated side. Robo3 staining at HH26 did not show any guidance defects in pre-crossing axons. Robo3 staining was performed on the same section as the Nkx2.2 staining. Scale bars = 50 µm.

Development • Supplementary information

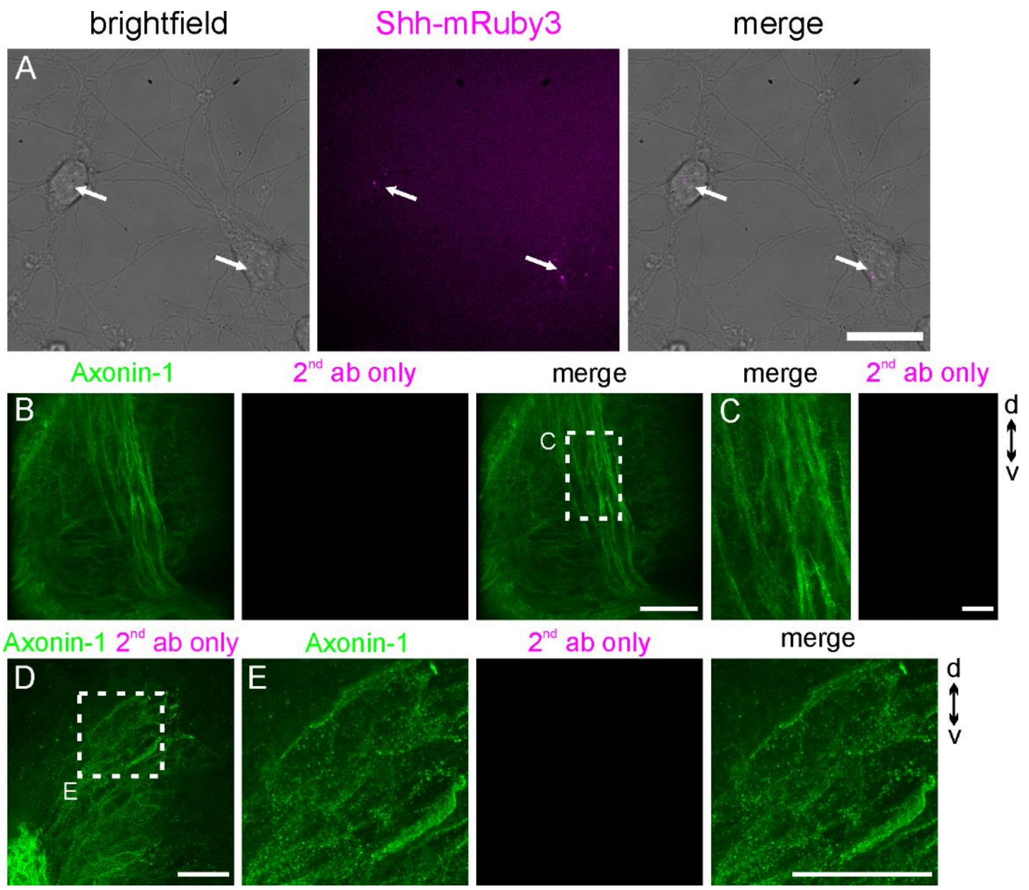

Fig. S10. Visualization of Shh *in vitro* and control staining *in vivo*.

(A) Live snapshot of cultured commissural neurons after 8DIV and overnight incubation with HEK cells expressing Shh-mRuby3 (magenta) in the axonal compartment of microfluidic chambers revealed that Shh-mRuby3 was transported along axons as it was enriched in the soma of neurons seen in the brightfield channel (arrows). (B-E) Control immunostaining (2nd antibody only) without primary antibody did not yield any significant signal in the ventral spinal cord (B-C) and in the dorsal spinal cord (D,E). Axonin-1 (green) was stained to visualize commissural axons and their soma. Acquisition settings were identical as for images shown in Fig. 9J-M. ab, antibody; d, dorsal; v, ventral. Scale bars: 20 µm (A,D,E), 50 µm (B), 10 µm (C).

Table S1. Source data

Available for download at
https://journals.biologists.com/dev/article-lookup/doi/10.1242/dev.202788#supplementary-data

Development • Supplementary information

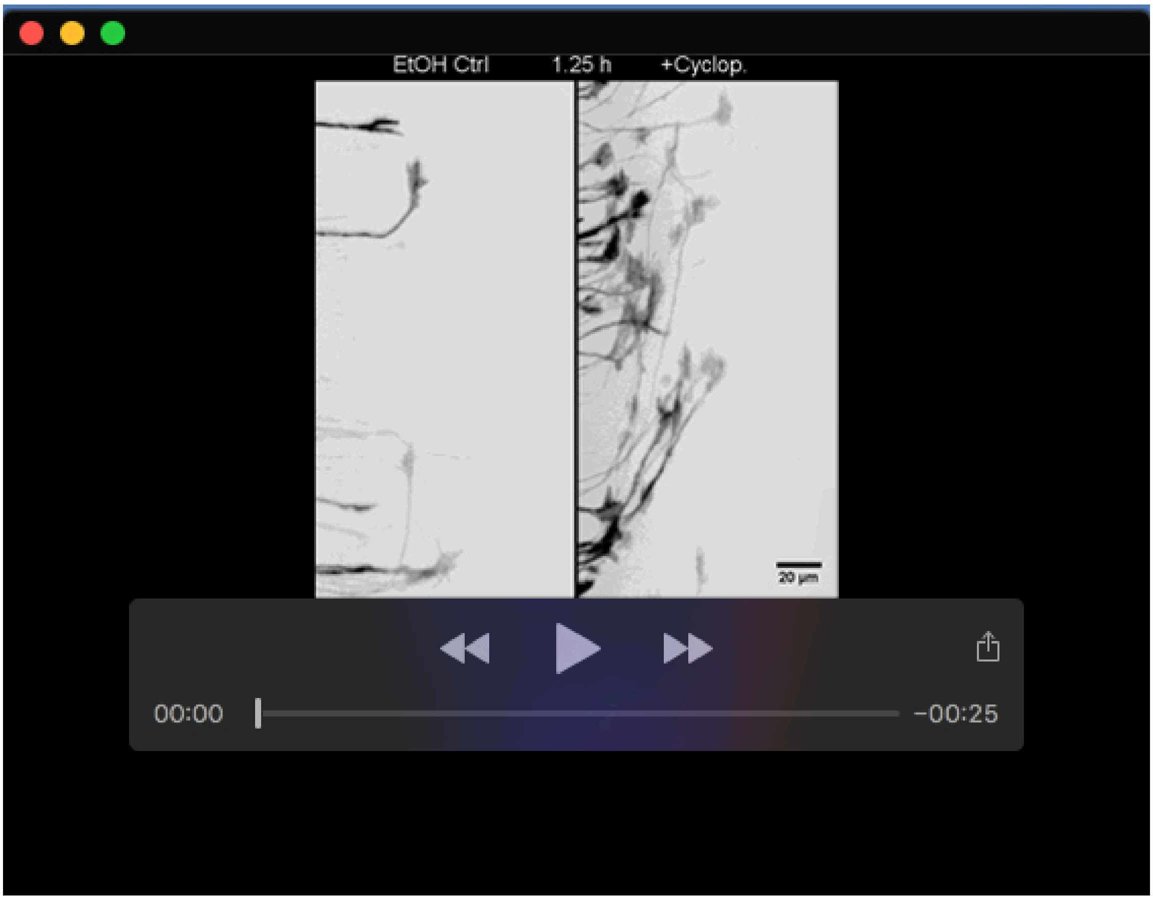

Movie 1. Cyclopamine-mediated inhibition of Smo induced aberrant dI1 axon guidance at the contralateral floorplate border.

Twenty-four hours of time-lapse recordings of the ventral midline of *ex vivo* spinal cords show Math1::tdTomato-F-positive dI1 axons (black) at the contralateral floorplate border in an ethanol-treated control (EtOH) or Cyclopamine-treated sample (Cyclop). dI1 axons turned rostrally in an organized manner in the control, but many of them showed aberrant trajectories in the presence of Cyclopamine. Maximum projections of z-stacks taken every 15 minutes. Rostral is up.

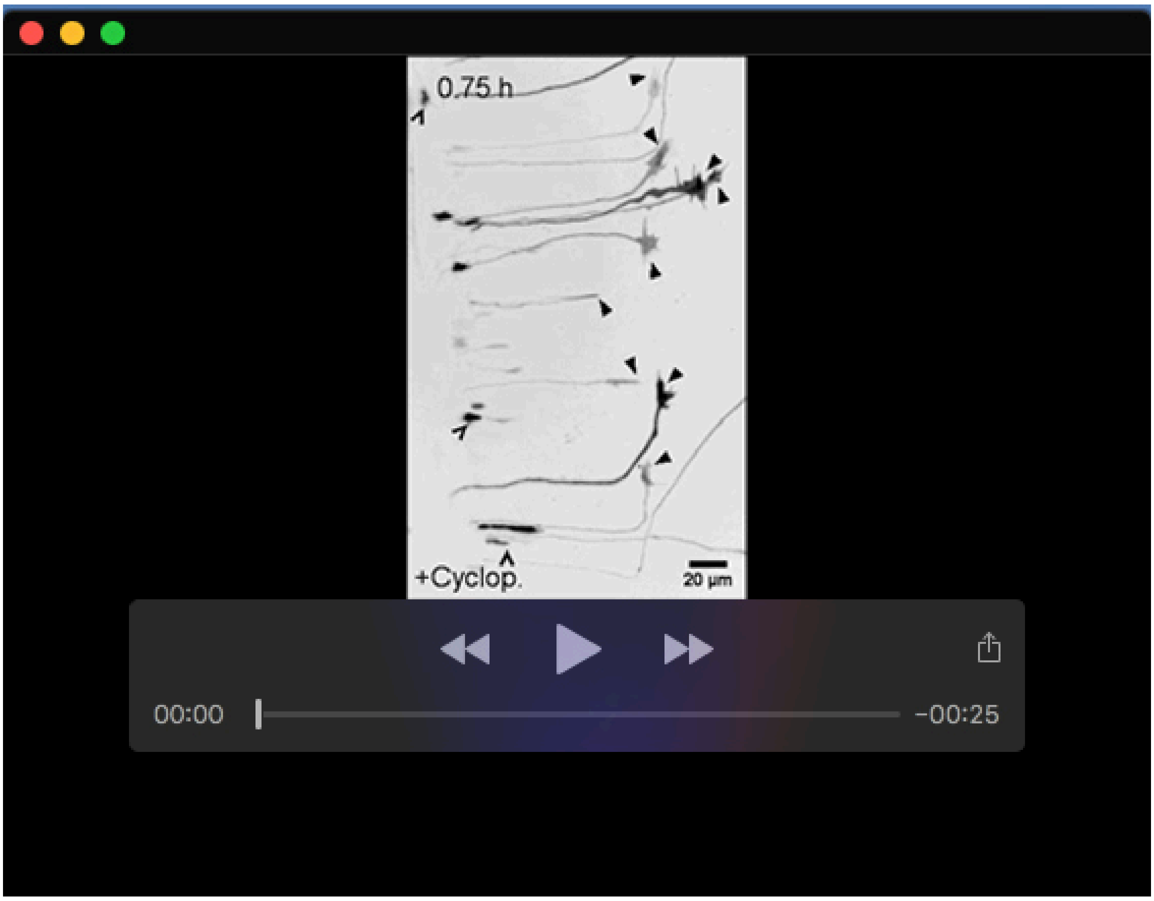

Movie 2. Cyclopamine-mediated inhibition of Smo induced aberrant dI1 axon guidance at the contralateral floorplate border only with a 4-hour delay.

Twenty-four hours of time-lapse recording of the ventral midline of *ex vivo* spinal cords shows Math1::tdTomato-F-positive dI1 axons (black) at the contralateral floorplate border in a Cyclopamine-treated sample (Cyclop.). Axons that were crossing or about to exit the floorplate (filled arrowheads) at the beginning of the recording/inhibition showed a normal trajectory with a rostral turn. However, most of the axons that were not yet in the floorplate when Cyclopamine was added showed aberrant navigation at the exit site (open arrowheads). Rostral is up.

Development • Supplementary information

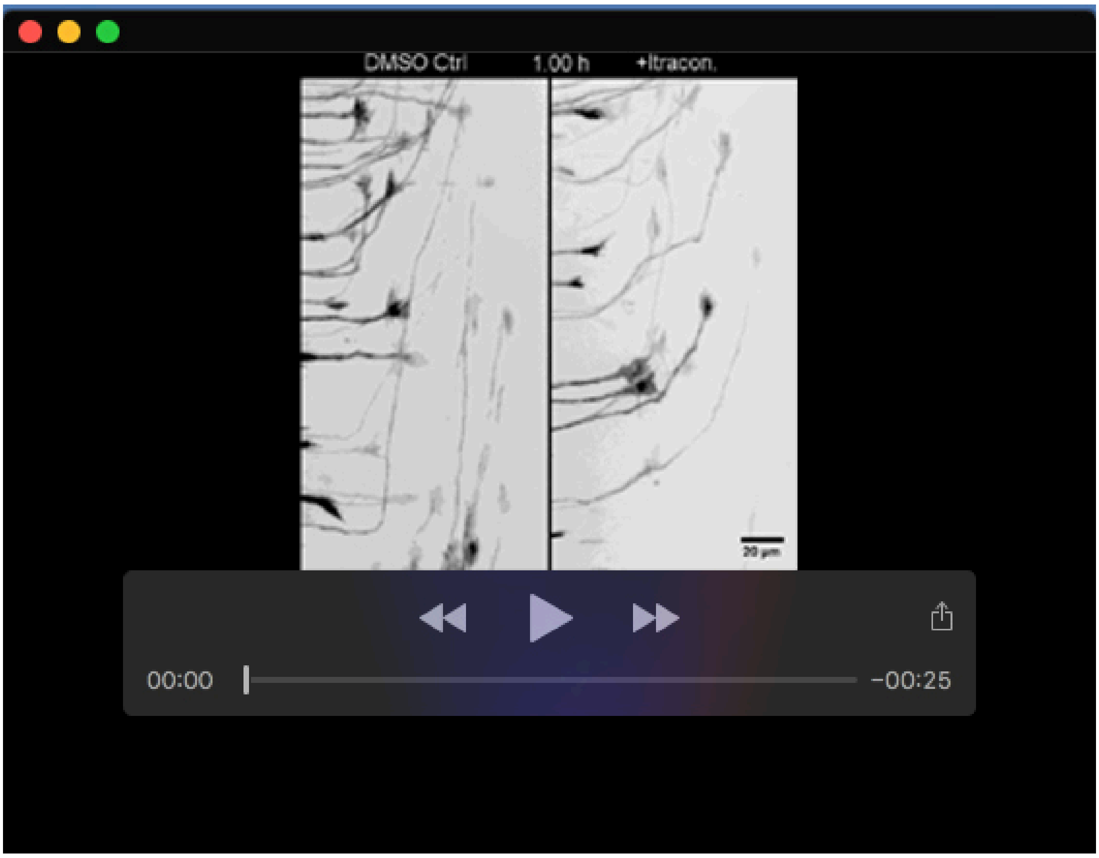

Movie 3. Blockade of Smo entry into the cilium with Itraconazole induced aberrant dI1 axon guidance at the contralateral floorplate border

Twenty-four hours of time-lapse recordings of the ventral midline of *ex vivo* spinal cords show Math1::tdTomato-F-positive dI1 axons (black) turn normally at the contralateral floorplate border in a DSMO-treated control. However, in the presence of Itraconazole (Itracon), dI1 axons showed an aberrant trajectory. Maximum projections of z-stacks taken every 15 minutes. Rostral is up.

Development • Supplementary information

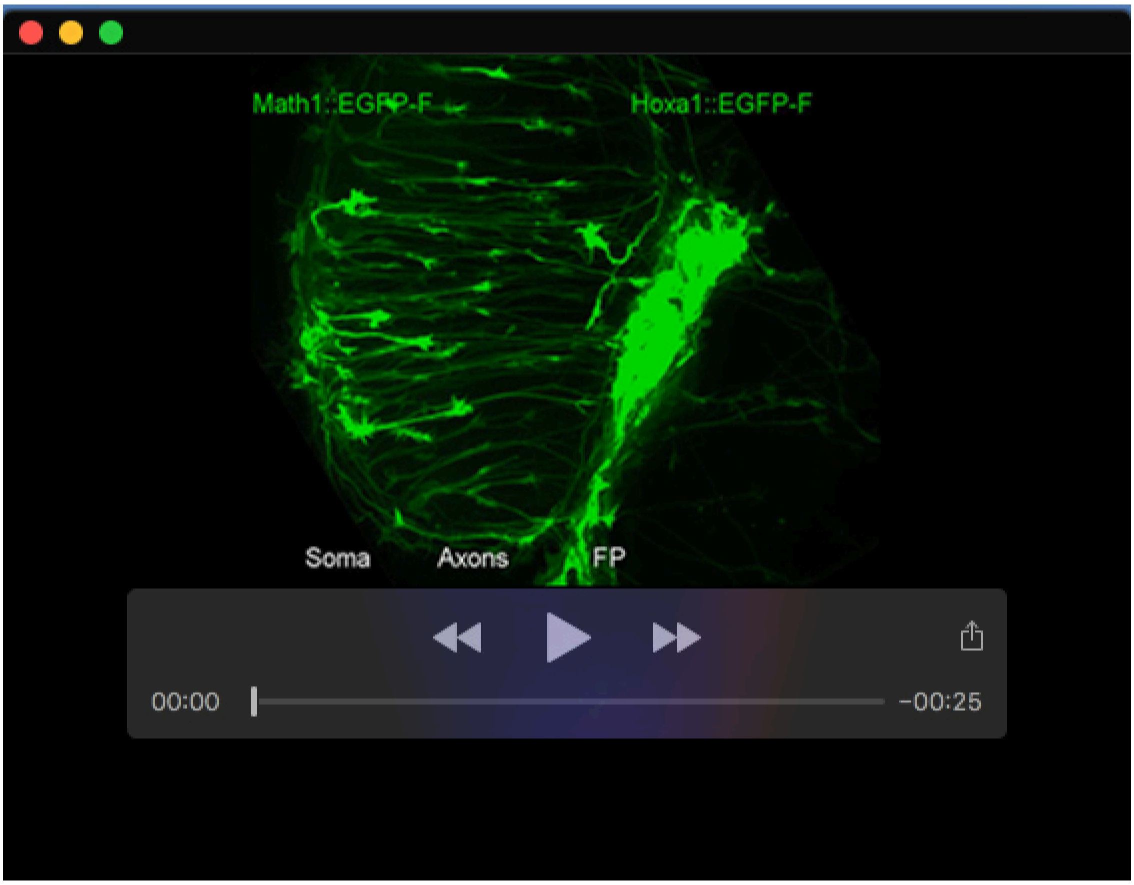

Movie 4. Shh-mRuby3-positive particles released by the floorplate move retrogradely along axons and reach the soma of dI1 neurons

150-min time-lapse sequence showing Shh-mRuby3-positive particles (magenta, white arrows) released from the floorplate, moving retrogradely along dI1 axons (magenta) and reaching the soma level of dI1 neurons in co-cultures of dorsal spinal cord- and floorplate explants. Maximum projections of z-stacks taken every 5 minutes. dI1 neurons were labelled by *in ovo* electroporation of Math1::EGFP-F and floorplate (FP) cells by Hoxa1::EGFP-F.

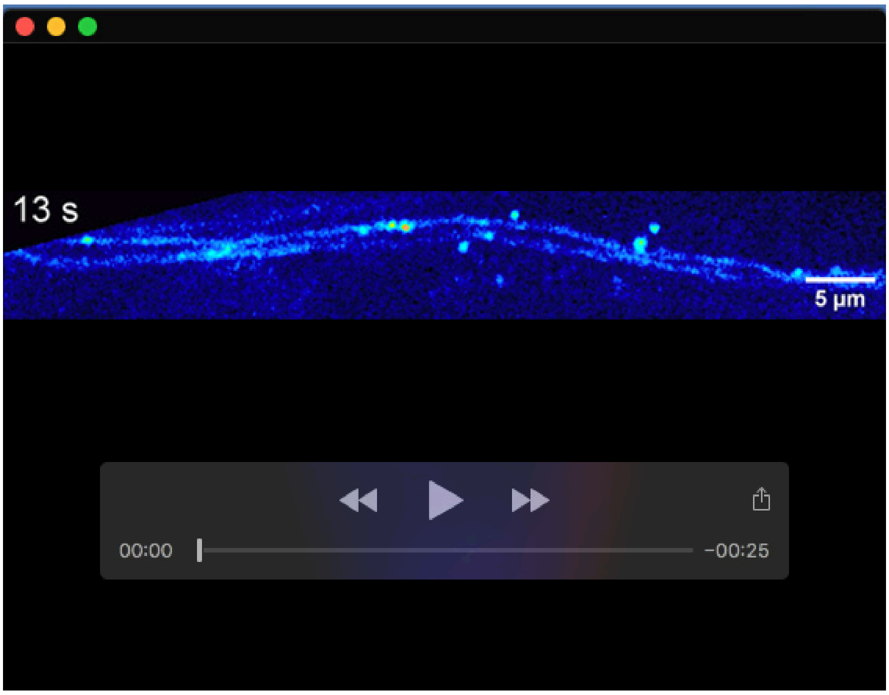

Movie 5. Shh is transported from the floorplate to the commissural neuron cell body in vitro. Time-lapse recording (1 image/s) of Shh-mRuby3-positive particles (pseudocolored) moving retrogradely along axons in explants (dorsal spinal cord and floorplate) co-cultured at a distance.

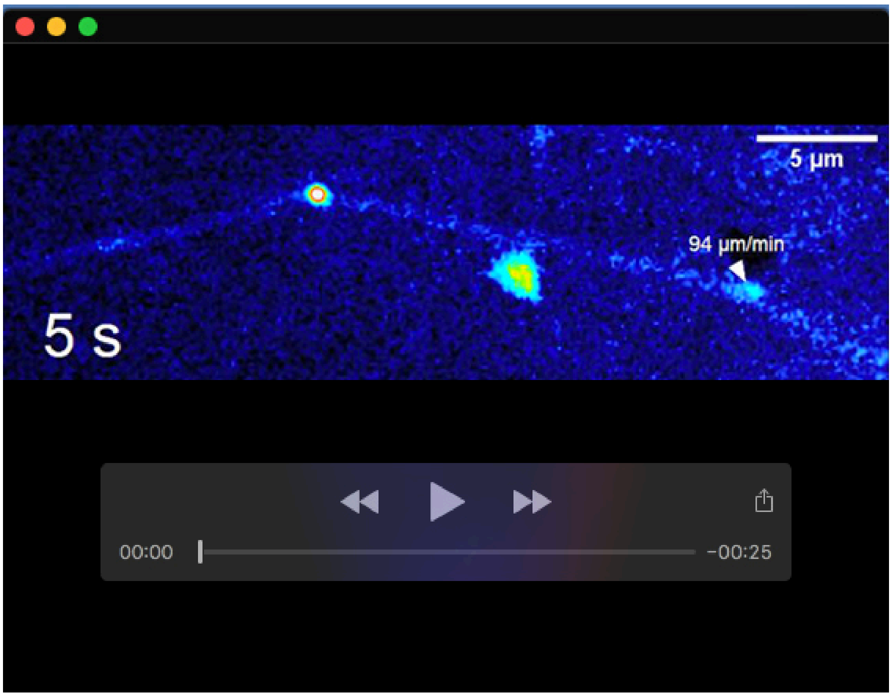

Movie 6. The speed of Shh transport is consistent with retrograde axonal transport. Time-lapse recording (1 image/s) of a Shh-mRuby3-positive particle (arrowheads, pseudocolored) moving retrogradely along axons in explants (dorsal spinal cord and floorplate) co-cultured at a distance. The average speed is given for each time point.
